# Retrospective Genomic Characterization of a 2017 Dengue Virus Outbreak, Burkina Faso

**DOI:** 10.3201/eid2806.212491

**Published:** 2022-06

**Authors:** Andrew G. Letizia, Catherine B. Pratt, Michael R. Wiley, Anne T. Fox, Mba Mosore, Bright Agbodzi, Clara Yeboah, Selassie Kumordjie, Nicholas Di Paola, Kone Cisse Assana, David Coulidiaty, Casimir Ouedraogo, Joseph H. Kofi Bonney, William Ampofo, Zékiba Tarnagda, Lassana Sangaré

**Affiliations:** Naval Medical Research Unit TWO, Singapore (A.G. Letizia);; University of Nebraska Medical Center, Omaha, Nebraska, USA (C.B. Pratt, M.R. Wiley);; Naval Medical Research Unit THREE, Ghana Detachment, Accra, Ghana (A.T. Fox);; Noguchi Memorial Institute for Medical Research, Accra (M. Mosore, B. Agbodzi, C. Yeboah, S. Kumordjie, J.H.K. Bonney, W. Ampofo);; US Army Medical Research Institute of Infectious Disease, Frederick, Maryland, USA (N. Di Paola);; Institut de Recherche en Sciences de la Santé, Bobo-Dioulasso, Burkina Faso (K.C. Assana, Z. Tarnagda);; Centre Hospitalier Universitaire Yalgado Ouédraogo, Ouagadougou, Burkina Faso (D. Coulidiaty, C. Ouedraogo, L. Sangaré)

**Keywords:** dengue virus, viruses, vector-borne infections, mosquito-borne diseases, outbreak, Burkina Faso, Africa, next-generation sequencing, diagnostics, RT-PCR, phylogenetics

## Abstract

Knowledge of contemporary genetic composition of dengue virus (DENV) in Africa is lacking. By using next-generation sequencing of samples from the 2017 DENV outbreak in Burkina Faso, we isolated 29 DENV genomes (5 serotype 1, 16 serotype 2 [DENV-2], and 8 serotype 3). Phylogenetic analysis demonstrated the endemic nature of DENV-2 in Burkina Faso. We noted discordant diagnostic results, probably related to genetic divergence between these genomes and the Trioplex PCR. Forward and reverse1 primers had a single mismatch when mapped to the DENV-2 genomes, probably explaining the insensitivity of the molecular test. Although we observed considerable homogeneity between the Dengvaxia and TetraVax-DV-TV003 vaccine strains as well as B cell epitopes compared with these genomes, we noted unique divergence. Continual surveillance of dengue virus in Africa is needed to clarify the ongoing novel evolutionary dynamics of circulating virus populations and support the development of effective diagnostic, therapeutic, and preventive countermeasures.

Dengue virus (DENV), the causative agent of dengue fever, is a mosquitoborne single-stranded RNA virus from the genus *Flavivirus*, often defined as 4 related serotypes (DENV-1, DENV-2, DENV-3, and DENV-4) (*1*). Globally, ≈4 billion persons in 128 countries are at risk for dengue fever (*2*). An estimated 390 million infections occur annually, of which 96 million are symptomatic (*3*), making DENV the most prevalent and rapidly spreading mosquitoborne viral disease of human beings (*4*). Clinical manifestations vary from a self-limited, potentially debilitating illness to hypovolemic shock; the mortality rate can be as high as 20% if left untreated (*4*).

An estimated 750 million persons are at risk for acquiring DENV in Africa, and the disease burden is estimated to be nearly equivalent to that of the Americas (*3*,*5*). Many countries in Africa lack a national surveillance system and reporting mechanism (*6*), causing dengue fever cases to be misdiagnosed as malaria (*7*), which might explain why among the 34 countries in Africa to report dengue fever, 12 were not reported by the country where it occurred but by travelers returning to their country of origin (*8*). Travel, particularly to Africa, is emerging as a well-recognized mechanism of intercontinental DENV spread (*9*,*10*).

Less than 1% of all global DENV envelope sequence data, key information for vaccine targets, come from isolates from Africa (*11*). A need exists for additional DENV sequencing, especially in Africa (*12*,*13*). The lack of genomic DENV data from Africa combined with complex transmission dynamics involving urban and sylvatic cycles impairs our understanding of DENV’s evolutionary history, transmission and spread (*13*), molecular diagnostics (*14*), antiviral targets (*15*), vector susceptibility (*16*), human immune response (*17*), vaccine development (*17*), and DENV spillover events (*18*). Determining which contemporary genotypes are in circulation is crucial to ensuring effective diagnostics and developing preventive and therapeutic countermeasures (*19*).

Burkina Faso, a country in West Africa with a population of ≈21 million persons, has had documented dengue fever outbreaks since 1925; known subsequent outbreaks occurred in 1982 and 2013 (*20*). In 2016, the World Health Organization declared an outbreak identifying 1,061 probable cases, primarily in the capital of Ouagadougou, population ≈2.5 million persons, in a setting of minimal surveillance and limited diagnostic ability (*21*). A larger outbreak, primarily in the central region that includes Ouagadougou, but involving all 13 health regions, occurred during August–November 2017, when Burkina Faso reported 9,029 suspected cases (*22*). Previous serotyping was conducted on 72 samples and demonstrated DENV-2 (58 cases), DENV-3 (12 cases), and DENV-1 (2 cases) (*23*); co-circulation of 3 serotypes occurred in Ouagadougou. The only published DENV genomes from either of these outbreaks were serotype 2, genotype Cosmopolitan, occurring after exposure during the 2016 outbreak among travelers returning to Japan and France (*24*,*25*).

By using in silico analyses, we determined whether unique DENV molecular divergence is occurring in Burkina Faso and assessed its impact on diagnostic assays and potential efficacy of vaccines and therapeutics. We sequenced DENV genomes from the 2017 outbreak in Burkina Faso to determine the molecular epidemiology of DENV and assess the homogeneity with targets for the US Centers for Disease Control and Prevention (CDC) Trioplex real-time reverse transcription PCR (RT-PCR), Dengvaxia (Sanofi Pasteur (https://www.sanofi.com) and TetraVax-DV-TV003 (Butantan Institute (http://butantan.gov.br) vaccine strains, and DENV antiviral epitopes.

## Methods

### Sample Processing and Sequencing

We obtained 791 deidentified human serum samples from patients with illness meeting the World Health Organization’s clinical case definition of dengue fever during the 2017 DENV outbreak in Burkina Faso (Appendix Table 1). Samples were provided by the Institut de Recherche en Sciences de la Santé (IRSS) in Bobo-Dioulasso and Centre Hospitalier Universitaire Yalgado Ouédraogo in Ouagadougou. We processed the samples at Noguchi Memorial Institute of Medical Research in Accra, Ghana.

We tested each sample by using molecular and serologic techniques, and if any test consistent with acute infection was positive, we selected that sample for genome sequencing (Appendix Figure 1). We conducted molecular-based evaluation for DENV by using the CDC Trioplex assay after extraction with QIAamp viral RNA mini kits (QIAGEN, https://www.qiagen.com) according to the manufacturer’s instructions. Serologic analyses included the detection of nonstructural protein 1 (NS1) antigen, DENV IgM, and DENV IgG (SD Bioline Dengue Duo; Abbott, https://www.globalpointofcare.abbott). We sequenced samples on an Illumina MiSeq (https://www.illumina.com) by using an enrichment-based method, as previously described, with modifications to enrich DENV (Appendix).

### Phylogenetics and Molecular Clock Analysis

To determine specific DENV genotypes, we aligned the Burkina Faso genomes with all complete genomes obtained from the US National Institutes of Health National Institute of Allergy and Infectious Diseases Virus Pathogen Database and Analysis Resource (http://www.viprbrc.org) and inferred a phylogenetic tree by using FastTree 2.1 (https://bioweb.pasteur.fr/packages/pack@FastTree@2.1.10). For our large-scale phylodynamics analysis, we retained all genomes from Africa and randomly subsampled ≈10% of the remaining genomes. We estimated time-calibrated phylogenies with the Markov chain Monte Carlo method implemented in BEAST 1.10.4 (https://beast.community) (Appendix). 

### Evaluation of PCR Diagnostics

We mapped primers and probe for the CDC Trioplex assay (patent no. WO2018169550A1), CDC DENV-1–4 RT-PCR (*26*), and Johnson et al. DENV RT-PCR (*27*) to the 29 Burkina Faso genomes in Geneious Prime 2021.0.3 (https://www.geneious.com). We then calculated mismatches within the primer–probe binding sites.

We further mapped the Trioplex forward primer, reverse1 primer, and probe sequences to an alignment of all available DENV genomes. We trimmed alignments to each primer–probe region and calculated the number of mismatches. We retained sequences with country information and calculated the proportion of genomes from each country with >1 mismatches. We represented these proportions in a chloropleth map by using ArcGIS Pro 2.8.0 (https://pro.arcgis.com).

### Vaccine and Epitope Analysis

We compared our Burkina Faso genomes to the Dengvaxia and TetraVax-DV-TV003 vaccine strains through sequence alignment in Geneious Prime 2021.0.3 by using MAFFT 7.427 (https://mafft.cbrc.jp/alignment/software). We were unable to obtain genome sequences of the TAK-003 dengue vaccine (Takeda, https://www.takeda.com). For the continental comparison, we downloaded all available DENV genomes from the Virus Pathogen Database and Analysis Resource and grouped them by serotype. We aligned the downloaded genomes to the vaccine strains with MAFFT and trimmed them to the membrane precursor (prM) and envelope (E) gene regions; we then retained and translated all genomes with country of origin. We assigned each represented country to a continent and calculated the proportion of sequences with divergent amino acids compared with the vaccines within each continental alignment.

We performed epitope mapping to compare the amino acid diversity of DENV strains from the 2017 outbreak in Burkina Faso to relevant epitopes that could serve as targets for antiviral human monoclonal antibodies. Appropriate epitopes for DENV-1–3 serotypes have been identified previously; we used an approach previously described comparing those amino acid targets and vaccine components to genomes from Burkina Faso (*28*) (Appendix).

### Data Availability

We submitted the consensus sequences that we generated from our Burkina Faso samples to GenBank (accession nos. MT261951–79). Probe sequences used during sequencing, nucleotide and amino acid alignments, and the .xml files are available online (https://github.com/cathrnbp/paper-dengue-2021).

### Ethics Considerations

The study protocol was approved by the Naval Medical Research Center’s Institutional Review Board (project no. NAMRU3.2018.0001). The study was in compliance with all applicable federal regulations governing the protection of human subjects.

## Results

### Dengue Virus Diversity in Burkina Faso

**Figure 1:**
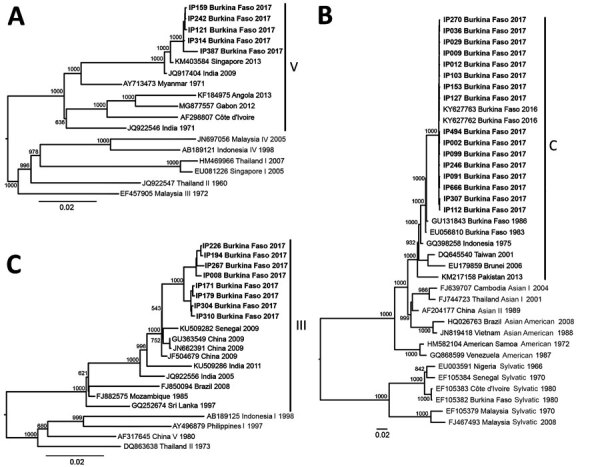
Phylogenetic trees of dengue virus (DENV) serotypes 1 (A), 2 (B), and 3 (C), inferred from an alignment of the 2017 Burkina Faso dengue virus outbreak genomes (boldface) and all other complete genomes from US National Institutes of Health National Institute of Allergy and Infectious Diseases Virus Pathogen Database and Analysis Resource (http://www.viprbrc.org) and pruned to representative genotypes. The Burkina Faso genomes were DENV-1 genotype V, DENV-2 genotype Cosmopolitan, and DENV-3 genotype III. GenBank accession numbers are provided for reference genomes.

Only 31 of the 791 samples had a measurable cycle threshold (Ct), and 20 of these met the criteria to be considered positive for the Trioplex assay (Appendix Table 1). Subsequent serologic tests detected NS1 antigen in 44 samples, DENV IgM in 18 samples, and DENV IgG in 27 samples, resulting in a total of 86 samples positive by PCR, NS1 antigen test, IgM test, or all 3 tests; many samples were positive by >1 test (Appendix Table, Figure 1).

We excluded samples positive only for DENV IgG. In total, we describe 29 DENV genomes with >85% coverage from 65 sequenced samples (Table). Genomic analysis confirmed the presence of serotypes 1–3; we identified no mixed serotype infections. To place these 29 genomes in context, we inferred maximum-likelihood and molecular-clock phylogenies for each serotype. Phylogenetic analysis of the genomes classified them into a single genotype for each serotype (Figure 1).

**Table:** Suspected dengue virus–positive samples from the 2017 Burkina Faso dengue virus outbreak, found to be positive by CDC Trioplex real-time RT-PCR or serologic testing, and sequencing results for samples that generated genomes with >85% coverage*

NMIMR laboratory ID	Specimen collection date, 2017	PCR results, Ct					Sequencing results		
			Serologic results				Serotype	Genome coverage, %	GenBank accession no.
			NS1 Ag	IgM	IgG				
IP-002	Oct 16	UND	+	–	–		DENV-2	99.6	MT261956
IP-008	Oct 16	35.5	+	–	–		DENV-3	99.1	MT261972
IP-009	Oct 16	37.1	+	–	–		DENV-2	99.7	MT261957
IP-012	Oct 16	40.6	+	–	–		DENV-2	87.6	MT261958
IP-029	Oct 17	37.5	+	–	–		DENV-2	98.8	MT261959
IP-036	Oct 17	40.6	+	–	–		DENV-2	99.5	MT261960
IP-091	Oct 26	36	+	–	–		DENV-2	99.8	MT261961
IP-099	Oct 25	40.3	+	–	–		DENV-2	99.6	MT261962
IP-103	Oct 25	34.6	+	–	–		DENV-2	99.7	MT261963
IP-112	Oct 24	39.5	+	–	–		DENV-2	99.7	MT261964
IP-121	Oct 23	29	+	–	–		DENV-1	99.7	MT261951
IP-127	Oct 23	38.2	–	–	–		DENV-2	99.4	MT261965
IP-153	Nov 8	33.7	+	–	–		DENV-2	99.8	MT261966
IP-159	Nov 9	32	+	–	–		DENV-1	99.3	MT261952
IP-171	Nov 9	33.8	+	+	+		DENV-3	94.3	MT261973
IP-179	Nov 13	22.5	+	–	–		DENV-3	94.4	MT261974
IP-194	Nov 17	25.2	–	–	–		DENV-3	99.7	MT261975
IP-226	Oct 4	25.4	–	–	–		DENV-3	99.8	MT261976
IP-242	Oct 9	29.5	–	–	–		DENV-1	99.6	MT261953
IP-246	Oct 9	31.1	–	–	–		DENV-2	99.8	MT261967
IP-267	Oct 12	19.3	–	–	–		DENV-3	99.8	MT261977
IP-270	Oct 12	37.1	–	–	–		DENV-2	99.5	MT261968
IP-304	Oct 27	24.1	+	–	–		DENV-3	99.7	MT261978
IP-307	Oct 30	37.3	–	–	–		DENV-2	99.7	MT261969
IP-310	Nov 2	30.3	+	–	+		DENV-3	95.7	MT261979
IP-314	Nov 2	23.8	+	–	–		DENV-1	99.7	MT261954
IP 387	Dec 12	UND	+	–	–		DENV-1	88.4	MT261955
IP 494	Nov 3	41.2	+	–	–		DENV-2	99.6	MT261970
IP 666	Nov 6	UND	+	–	–		DENV-2	99.6	MT261971

*CDC, US Centers for Disease Control Prevention; Ct, cycle threshold; NMIMR, Noguchi Memorial Institute for Medical Research; NS1 Ag, nonstructural protein 1 antigen; RT-PCR, reverse transcription PCR; UND, undetected; +, positive; –, negative.

**Figure 2:**
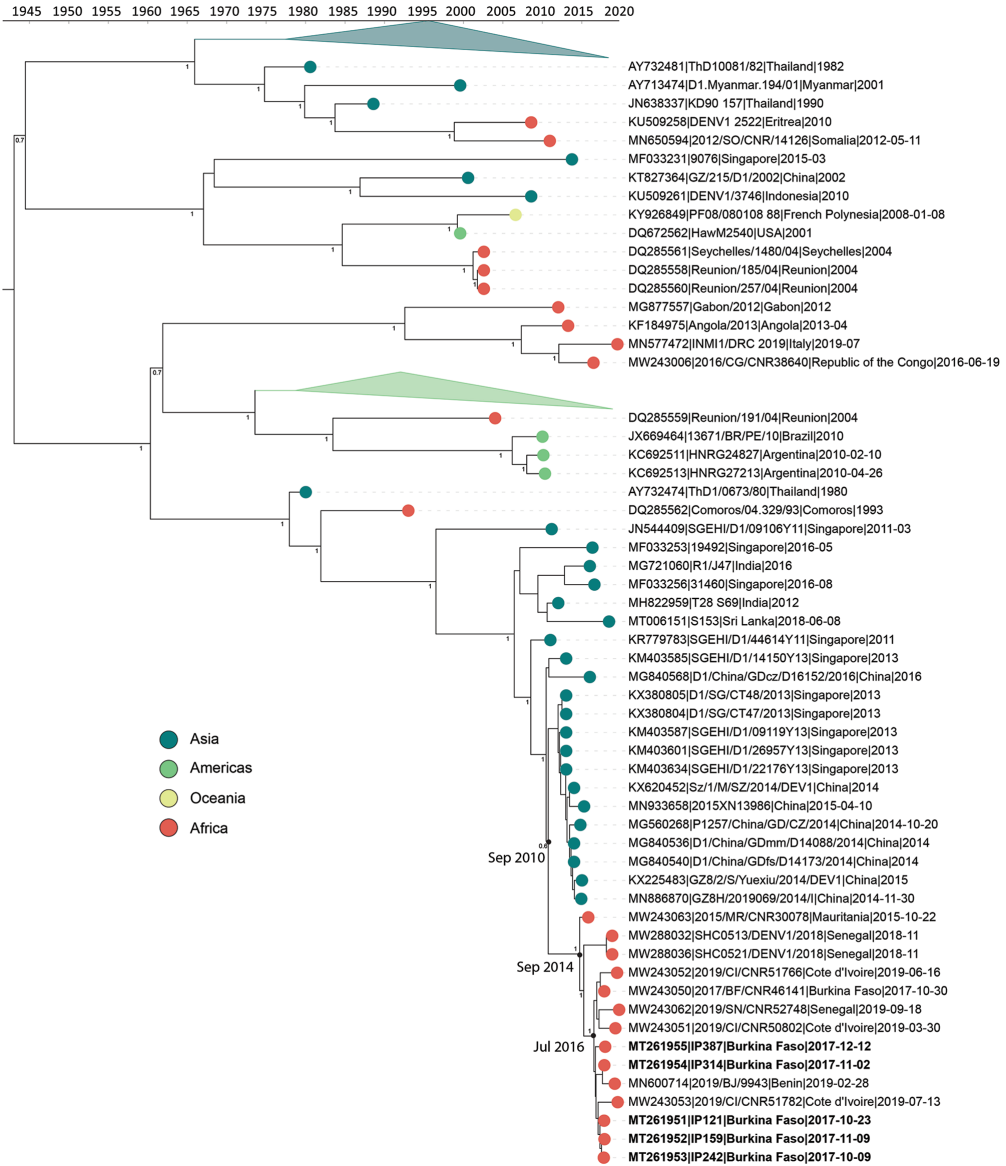
Time-calibrated phylogenetic trees of a subset of global dengue virus 1 genomes and 2017 Burkina Faso dengue virus outbreak genomes (boldface). Colored circles indicate geographic origin. Dates indicate the most recent common ancestor for the 2017 Burkina Faso dengue virus outbreak and all genomes from Africa. Posterior probabilities are indicated at major nodes. GenBank accession numbers are provided for reference genomes.

We sequenced 5 DENV-1 genotype V, 16 DENV-2 Cosmopolitan, and 8 DENV-3 genotype III genomes. The DENV-1 genomes grouped closely with a traveler from France returning from Benin in 2019 (GenBank accession no. MN600714) (*29*) and the DENV-2 genomes with a traveler returning to France from Burkina Faso in 2016 (GenBank accession nos. KY627762/3). The DENV-1 genomes have a most recent common ancestor (MRCA) from July 2016 (95% highest posterior density [HPD] 2016.1–2016.9) (Figure 2) and form a monophyletic clade with other genomes from West Africa sampled during 2015–2019, having a common ancestor from September 2014 (95% HPD 2014.0–2015.3). Our analysis of all complete Africa DENV-1 genomes indicates multiple separate introductions into Africa, followed by localized spread (Figure 2). DENV-1 may have been introduced into West Africa as early as 2010 (95% HPD 2009.9–2011.4), probably from Asia. The phylogenetic tree inferred from all E gene sequences corroborates this conclusion (Appendix Figure 2).

**Figure 3:**
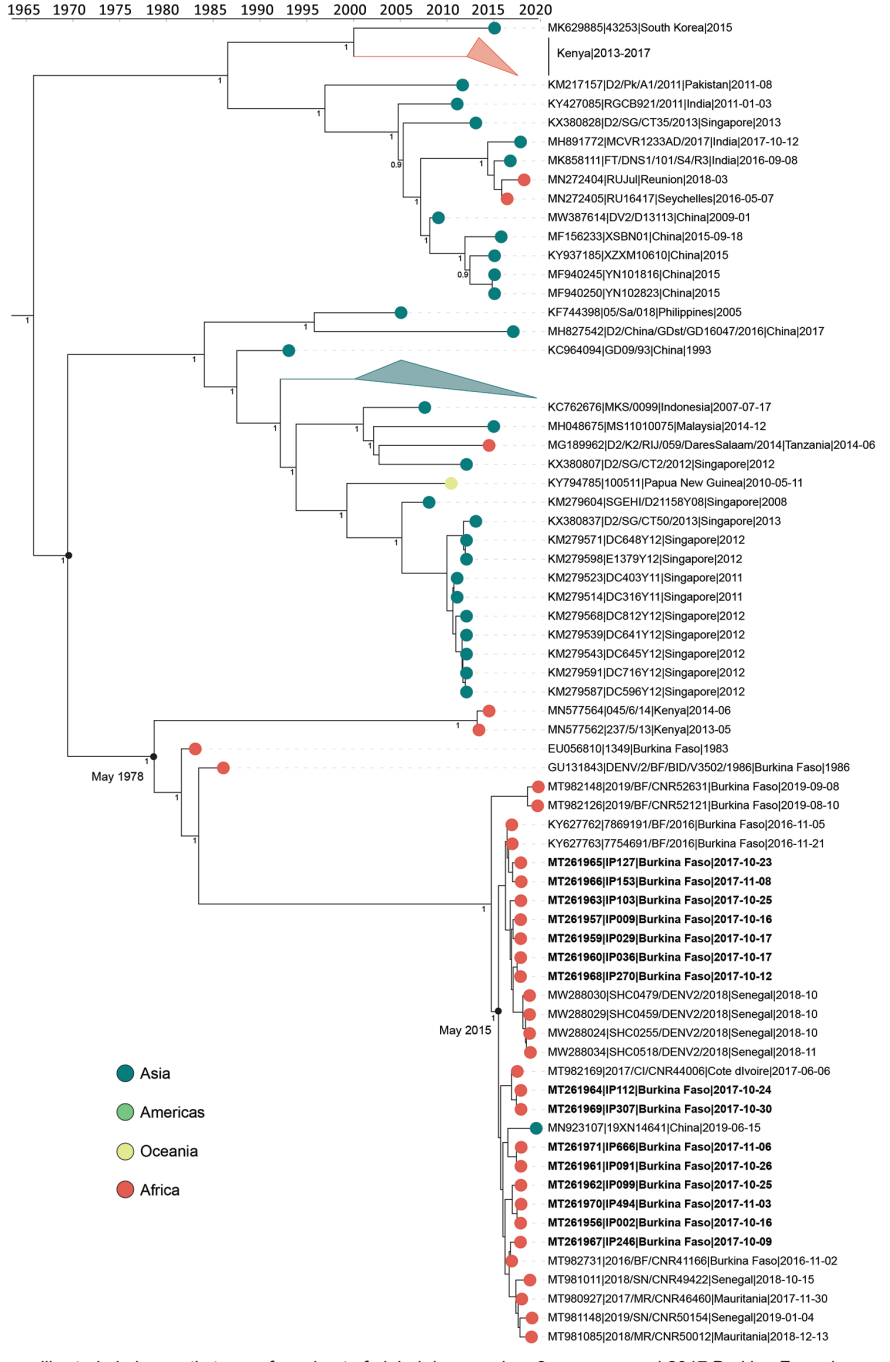
Time-calibrated phylogenetic trees of a subset of global dengue virus 2 genomes and 2017 Burkina Faso dengue virus outbreak genomes (boldface). Colored circles indicate geographic origin. Dates indicate the most recent common ancestor for the 2017 Burkina Faso dengue virus outbreak and all genomes from Africa. Posterior probabilities are indicated at major nodes. GenBank accession numbers are provided for reference genomes.

Our DENV-2 genomes form several clusters across a monophyletic Africa clade with a MRCA from May 2015 (95% HPD 2014.8–2015.9) (Figure 3). DENV-2 genomes in this clade have been sequenced from countries across West Africa, and available data suggest the 2017 Burkina Faso variant was probably exported to China (Figure 3), demonstrating the movement of DENV from Africa to Asia. In contrast to DENV-1, DENV-2 genomes share a common ancestor with other genomes from Burkina Faso collected as far back as 1983. The MRCA of the entire monophyletic Africa clade, including 2 outlying genomes from Kenya, was from May 1978 (95% HPD 1975.3–1981.1). The long branch from the early 1980s to 2015 is probably the result of undersampling rather than the absence of human DENV-2 cases. To ensure this long branch was not a result of excluded sequencing data in our complete genome analysis, we inferred phylogenetic trees from all E gene sequences from partial and complete genomes (Appendix Figure 3). We identified partial genomes from an additional 9 Africa countries that clustered within the same clade as these Burkina Faso genomes; only genomes sampled from Indonesia in the 1970s were antecedent. These data demonstrate that DENV-2 has been circulating across Africa since the late 1970s, when it was probably introduced from Southeast Asia.

**Figure 4:**
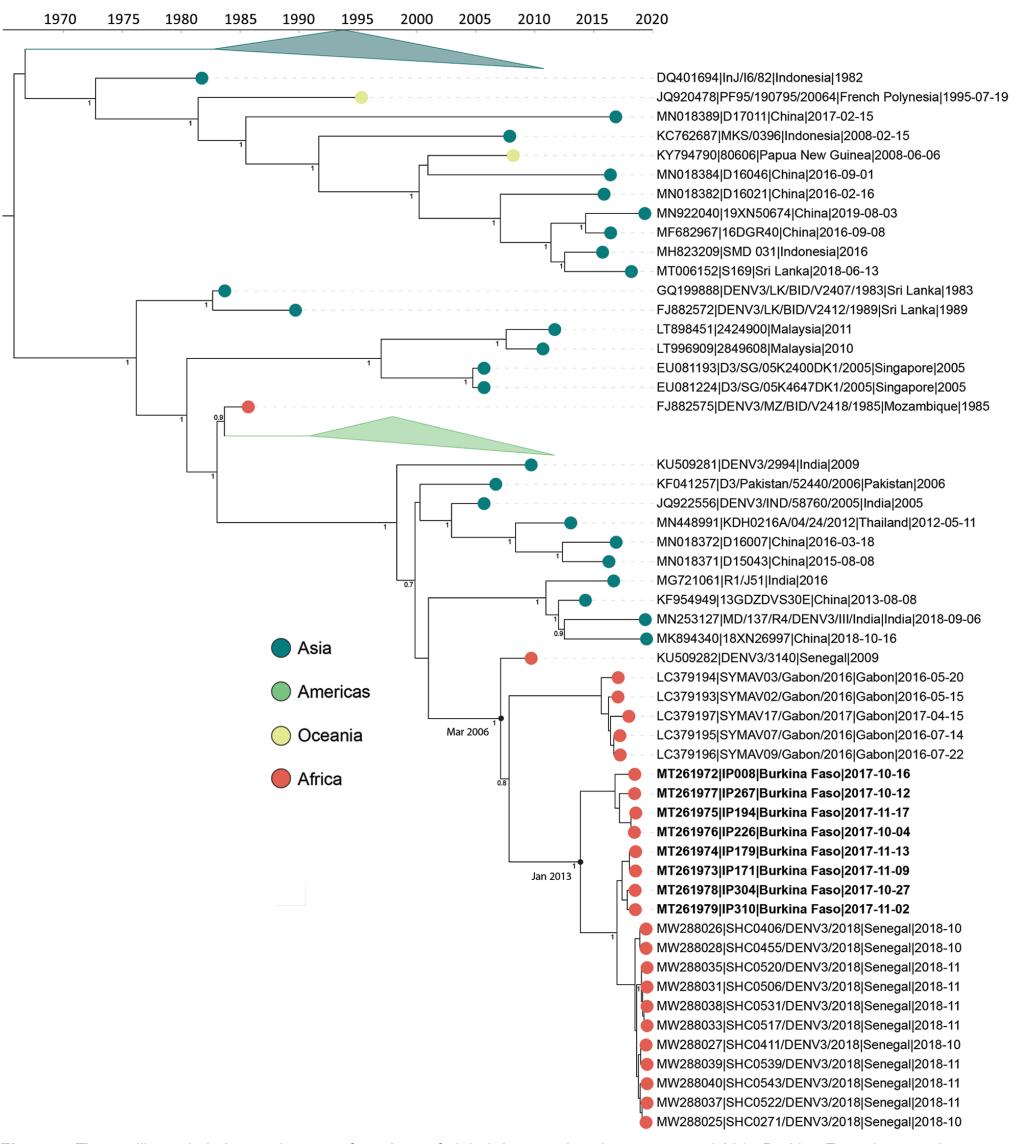
Time-calibrated phylogenetic trees of a subset of global dengue virus 3 genomes and 2017 Burkina Faso dengue virus outbreak genomes indicated (boldface). Colored circles indicate geographic origin. Dates indicate the most recent common ancestor for the 2017 Burkina Faso dengue virus outbreak and all genomes from Africa. Posterior probabilities are indicated at major nodes. GenBank accession numbers are provided for reference genomes.

The molecular-clock phylogeny for DENV-3 genomes from Burkina Faso cluster into 2 distinct clades within a monophyletic Africa clade (Figure 4). The MRCA for the DENV-3 Burkina Faso clade was from January 2013 (95% HPD 2010.8–2014.9) and the MRCA of all Africa genomes from March 2006 (95% HPD 2004.0–2008.1); these genomes were probably introduced from Asia. When including all E gene genomes in a phylogenetic analysis, we see introductions to 8 additional countries in Africa (Appendix Figure 4). These results provide evidence of widespread dengue virus circulation within Africa with DENV-1 existing for >7 years, DENV-2 for >39 years, and DENV-3 for >11 years.

### Trioplex Assay in Africa

Although only 31 of the 791 samples we tested were positive by the Trioplex assay, after sequencing we unexpectedly gained complete genomes from 3 samples that were negative by PCR, indicating concerns with PCR sensitivity. The median Trioplex assay Ct value for DENV-1 genomes was 29.5, for DENV-2 was 37.9, and DENV-3 25.3 (Appendix Figure 5), suggesting that the Trioplex assay was less sensitive against DENV-2 than DENV-1 and DENV-3. This finding is corroborated by the limits of detection reported in the Trioplex package insert, which are stated as 5.82 × 10^4^ genome copies/mL for DENV-1, 8.25 × 10^4^ genome copies/mL for DENV-2, and 4.36 × 10^4^ genome copies/mL for DENV-3.

In addition, we performed an in silico analysis of these assays by mapping the primers and probe to the Burkina Faso genomes and comparing nucleotide homogeneity. The Trioplex primers and probe were identical to the DENV-1 and DENV-3 Burkina Faso genomes, but both the forward and reverse1 primers had a single mismatch when mapped to the DENV-2 genomes. We also investigated the CDC DENV-1–4 RT-PCR (*26*), which had 5 mismatches across the primers and probe for the DENV-1, DENV-2, and DENV-3, and the Johnson et al. RT-PCR (*27*), which had 8 mismatches (Appendix Figure 6).

**Figure 5:**
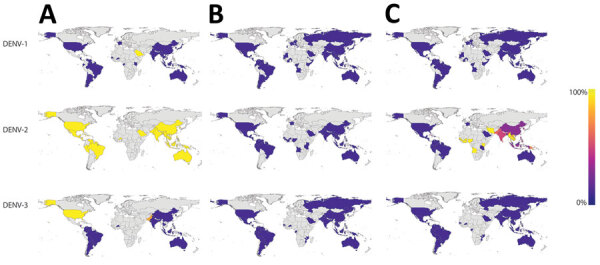
Nucleotide identity between dengue virus molecular diagnostics and all sequenced DENV genomes from the 2017 Burkina Faso dengue outbreak. The map indicates the proportion of genomes from each country with >1 mismatches against the Trioplex PCR forward primer (A), probe (B), and reverse1 primer (C). Countries in gray have no data. DENV-1 and DENV-3 have concordant nucleotide identity to the primers and probe, but most DENV-2 forward primer and reverse1 primer in sequences from Africa have a high proportion of genomes with >1 mismatches to the Trioplex PCR’s primers and probe. DENV-1, dengue virus serotype 1; DENV-2, dengue virus serotype 2; DENV-3, dengue virus serotype 3

To determine if these mismatches were specific to Burkina Faso or indicated a more global problem, we mapped the Trioplex primers and probe to all available DENV genomes and calculated the proportion of genomes from each country that exhibited <100% homogeneity to the primers and probe (i.e., had >1 mismatch) (Figure 5). Because the Trioplex assay targets the 5′ untranslated region and many genomes lacked coverage in this region, especially for the forward primer, they could not be included. For DENV-1 and DENV-3, we observed almost complete homogeneity between the probe and reverse1 primer within all countries. The forward primer was similarly identical, except for some divergence in Asia and North America. Conversely, for DENV-2, although the probe sequence was almost completely identical to the DENV-2 genomes at its binding site, the forward primer exhibited a single mismatch in every genome included in our analysis. This mismatch is likely the cause of the lowered limit of detection for DENV-2 compared with DENV-1 and DENV- 3, as noted previously. Approximately 95% of genomes from Africa had >1 mismatches in the reverse1 primer (and a mismatch in the forward primer) compared with 6% of genomes from South America, 20% from Oceania, and 50% from Asia (Figure 5).

### Dengue Vaccines and African Variants 

**Figure 6:**
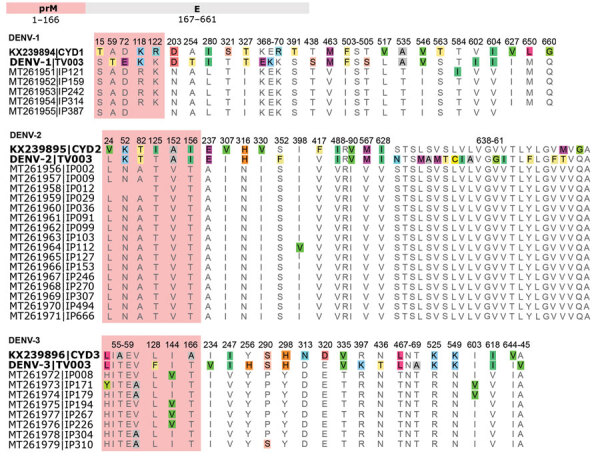
Dengue virus prM and E protein sequence alignments of Dengvaxia and TetraVax-DV-TV003 vaccine strains (boldface) and 2017 Burkina Faso dengue virus outbreak genomes for serotypes 1, 2, and 3. Only amino acid positions with disagreements are shown; single-point disagreements are highlighted. For clarity, prM protein sequences are shaded in red. Numerals represent the prM and E protein amino acid position. CYD, Dengvaxia vaccine; DENV-1, dengue virus serotype 1; DENV-2, dengue virus serotype 2; DENV-3, dengue virus serotype 3; E, envelope; prM, premembrane; TV003, TetraVax-DV-TV003 vaccine.

The 29 full genomes from the Burkina Faso 2017 outbreak were compared with the Dengvaxia and TetraVax-DV-TV003 vaccine strains for each serotype (Figure 6). Dengvaxia is based on an immunoprotective serotype-specific prM and E gene region in a background of yellow fever virus while TetraVax-DV-TV003 uses a different dengue virus serotype. Therefore, the comparison with the full genome sequences focused on the prM and E proteins. Divergent amino acids occurred throughout the prM and E proteins between the vaccine strain and Burkina Faso wild types, including 20 substitutions for DENV-1 sequences, 18 for DENV-2, and 17 for DENV-3 when compared with the Dengvaxia vaccine and 18 substitutions for DENV-1, 25 for DENV-2, and 19 for DENV-3 when compared with the TetraVax-DV-TV003 vaccine. None of the discordant amino acids clustered to any particular structural domain.

**Figure 7:**
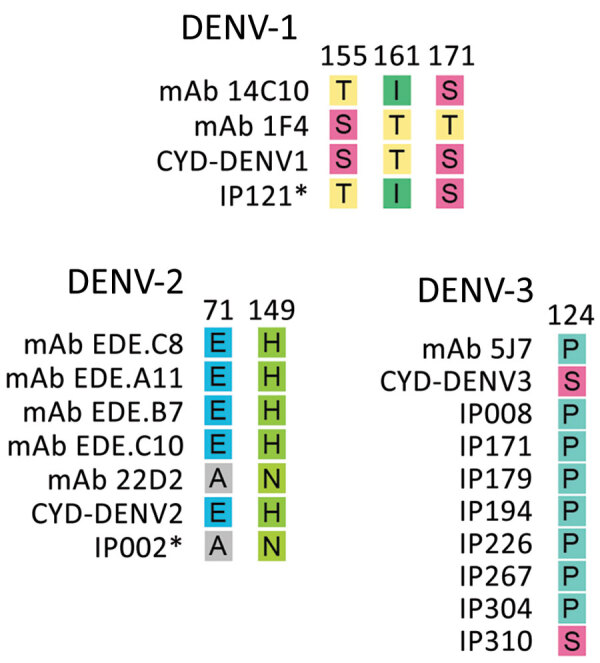
Amino acid mismatch comparison between 2017 Burkina Faso dengue virus outbreak genomes and virus neutralizing human mAbs for the 3 dengue virus serotypes. The amino acid changes presented are expected to disrupt binding between the envelope protein and heavy chain of the monoclonal antibodies. Dengvaxia vaccine amino acid included for comparison. Asterisk indicates all of the 2017 Burkina Faso dengue virus outbreak genomes share the same amino acid at that position. Numerals represent the E protein amino acid position. CYD, Dengvaxia vaccine; DENV-1, dengue virus serotype 1; DENV-2, dengue virus serotype 2; DENV-3, dengue virus serotype 3; E, envelope; mAb, monoclonal antibody.

We compared the Burkina Faso wild type virus sequences with the vaccine strains at 8 B cell epitopes (Figure 7). The noted divergence is similar to that seen in Southeast Asia and the Americas and has been previously described at E protein sites 155, 161, and 171 for DENV-1; sites 71 and 149 for DENV-2; and site 124 for DENV-3 (*28*).

Because of the paucity of genomic data from Burkina Faso, we expanded our analysis to the continental scale. We calculated the proportion of genomes within each continental alignment diverging from the vaccine sequence at each amino acid position. Amino acid positions with >5% divergence from the Dengvaxia (Appendix Figure 7) and TetraVax-DV-TV003 (Appendix Figure 8) vaccine strains were retained. In a minimum of 12 amino acid positions across each serotype and vaccine comparison, DENV genomes from Africa had the greatest proportion of genomes divergent from the vaccine strains. DENV genomes circulating in Africa exhibit their own genomic diversity, impairing the potential effectiveness of a DENV vaccine on that continent.

## Discussion

We sequenced 29 full DENV genomes from the 2017 outbreak in Burkina Faso, confirming cocirculation of DENV-1, DENV-2, and DENV-3 serotypes. Phylogenetic analysis of DENV-2 genomes show the most similar genomes to those from the DENV 2017 outbreak are also from Burkina Faso, dating from 1983 through 1986. The genetic similarities between DENV-2 strains from 2017 and those from >30 years ago suggest local circulation of DENV-2 genotype Cosmopolitan both within Burkina Faso and in other countries in West Africa and that DENV-2 is endemic to this area. All the genomes from the 2017 outbreak in Burkina Faso were most closely related to strains from Africa or Asia and not those from the Americas. This finding could be attributable to greater trade, travel, and economic-based contact between Burkina Faso and other countries of Africa with Asia as opposed to countries in the Americas.

We obtained 2 complete genomes and 1 partial genome from PCR-negative samples, and the Ct for DENV-2 samples was consistently higher than that for DENV-1 and DENV-3, suggesting a drop in assay sensitivity against DENV-2 genomes. This decrease is probably because of mismatches between the primers and probe and target sequences, or because the samples were too degraded for PCR but not for hybrid capture sequencing, which seems unlikely. An in silico analysis identified mismatches between the primers and probe for the Trioplex assay and DENV-2 genomes, both in our Burkina Faso genomes and across Africa. The Trioplex assay was designed during the 2015–2016 Zika virus epidemic to differentiate between Zika, chikungunya, and DENV infections and has also been made available to international laboratories in a lyophilized format at no charge (*30*). This altruism means that it is a commonly used assay in low-resource laboratories, such as those in many countries in Africa. The Trioplex assay was validated by using samples collected in Puerto Rico (*30*). In our analyses, genomes from the Americas were most congruent with the Trioplex primers and probe and those from Africa were the least congruent. Further, the target of the Trioplex assay is near the 5′ untranslated region and vulnerable to degradation, which is more likely to occur in low-resource countries, where samples are often transported to a central laboratory under less than ideal conditions for RNA preservation. The CDC developed another PCR with serotype-specific primers and probe, the CDC-DENV-1–4 RT-PCR (*26*), based on the Johnson et al. RT-PCR (*27*), but both of these assays exhibited even less nucleotide homogeneity in silico than the Trioplex assay. The observed genomic divergence, discordance between sequencing and PCR results, and existence of multiple mismatches in the primer binding site within samples from Africa suggest that Africa-specific virus evolution is occurring, probably leading to an underreporting of dengue cases because of insensitive diagnostics. This probability necessitates the development of diagnostics that account for the unique molecular divergence occurring in Africa to have an accurate assessment of the disease burden of DENV and improve patient care.

Because of the threat that DENV poses to Africa, the number of outbreaks, and the lack of countermeasures, it is not too early to consider preventive measures. The Burkina Faso genomes enabled us to perform in silico analyses of DENV vaccine efficacy and assess divergence from known important epitopes. In general, the 3 DENV serotypes circulating during the 2017 outbreak in Burkina Faso were very similar to the vaccine strains used in the CYD-Dengvaxia and TetraVax-DV-TV003 vaccines. Although the Dengvaxia vaccine was noted to have decreased efficacy against DENV-2 compared with other serotypes (*31*), it appears to have been more efficacious against the DENV-2 Cosmopolitan genotype than against the Asian 1 genotype (*28*). However, there were key positions in the Dengvaxia and TetraVax-DV-TV003 vaccine sequences where genomes from Africa diverged more often than genomes from other continents, indicating the development of unique diversity within Africa. Further research is needed to understand how various genotypes and subtle differences at the amino acid level of prM and E proteins affect clinical immunity. Additional in vivo testing is necessary to determine if a dengue vaccine could be used in West Africa.

The amino acid prM and E protein sequences from the Burkina Faso DENV outbreak were also very similar to known targets for B cell epitopes. The differences noted have been previously reported in DENV strains from the Americas and Southeast Asia (*28*). However, we observed 2 mismatches at important epitope sites E71 and E149 among all DENV-2 Cosmopolitan samples. Although this discordance is documented in other DENV-2 genotypes, including American, American-Asian, Asian 1, and Asian II genotypes, it is not as well defined in the Cosmopolitan genotype.

A limitation of our study is that >1 year had passed since the initial collection of the samples before next-generation sequencing was performed, introducing multiple factors that could have contributed to this low percentage of positive results: sample degradation over time, less than ideal storage, low viremia, poor coverage of the assay, or a combination of these factors. Using further molecular diagnostics may have revealed more DENV-positive samples but were not available in the country at the time of the study. Additional genomes could have increased the probability of detecting unusual genomes or amino acid changes. Assessing the evolutionary patterns of DENV is difficult because so few whole DENV genomes from Africa are available on GenBank to compare with the genomes from Burkina Faso. Finally, donor virus strains other than Dengvaxia and TetraVax-DV-TV003 were not assessed.

Our assessment of DENV whole genomes from Burkina Faso provide information on the molecular epidemiology of this virus and divergence from diagnostics, vaccine strains, and B cell epitopes. Further surveillance of contemporary DENV genotypes in Africa is needed to address the contemporary antigenic and genetic variations within a region. The endemicity of DENV and increasing number of outbreaks in countries like Burkina Faso suggest the need for the development of diagnostics that account for ongoing viral evolution in Africa and consideration for adding countries in Africa to DENV clinical trials to address the emerging public health threat.

Appendix Additional information about retrospective genomic characterization of a 2017 dengue virus outbreak, Burkina Faso.

## References

[1] Simmons CP, Farrar JJ, Nguyen V, Wills B. Dengue. N Engl J Med. 2012;366:1423–32. 10.1056/NEJMra1110265 22494122

[2] Brady OJ, Gething PW, Bhatt S, Messina JP, Brownstein JS, Hoen AG, et al. Refining the global spatial limits of dengue virus transmission by evidence-based consensus. PLoS Negl Trop Dis. 2012;6:e1760. 10.1371/journal.pntd.0001760 22880140 PMC3413714

[3] Bhatt S, Gething PW, Brady OJ, Messina JP, Farlow AW, Moyes CL, et al. The global distribution and burden of dengue. Nature. 2013;496:504–7. 10.1038/nature12060 23563266 PMC3651993

[4] Guzman MG, Harris E. Dengue. Lancet. 2015;385:453–65. 10.1016/S0140-6736(14)60572-9 25230594

[5] Weetman D, Kamgang B, Badolo A, Moyes CL, Shearer FM, Coulibaly M, et al. *Aedes* mosquitoes and *Aedes*-borne arboviruses in Africa: current and future threats. Int J Environ Res Public Health. 2018;15:E220. 10.3390/ijerph15020220 29382107 PMC5858289

[6] Lim JK, Carabali M, Lee JS, Lee KS, Namkung S, Lim SK, et al. Evaluating dengue burden in Africa in passive fever surveillance and seroprevalence studies: protocol of field studies of the Dengue Vaccine Initiative. BMJ Open. 2018;8:e017673. 10.1136/bmjopen-2017-017673 29358421 PMC5780679

[7] Stoler J, Al Dashti R, Anto F, Fobil JN, Awandare GA. Deconstructing “malaria”: West Africa as the next front for dengue fever surveillance and control. Acta Trop. 2014;134:58–65. 10.1016/j.actatropica.2014.02.017 24613157

[8] Fournet F, Rican S, Vaillant Z, Roudot A, Meunier-Nikiema A, Kassié D, et al. The influence of urbanization modes on the spatial circulation of flaviviruses within Ouagadougou (Burkina Faso). Int J Environ Res Public Health. 2016;13:E1226. 10.3390/ijerph13121226 27973402 PMC5201367

[9] Schwartz E, Meltzer E, Mendelson M, Tooke A, Steiner F, Gautret P, et al. Detection on four continents of dengue fever cases related to an ongoing outbreak in Luanda, Angola, March to May 2013. Euro Surveill. 2013;18:20488. 10.2807/ese.18.21.20488-en 23725977

[10] Toro C, Trevisi P, López-Quintana B, Amor A, Iglesias N, Subirats M, et al. Imported dengue infection in a Spanish hospital with a high proportion of travelers from Africa: a 9-year retrospective study. Am J Trop Med Hyg. 2017;96:701–7. 10.4269/ajtmh.16-0335 28167601 PMC5361549

[11] Pollett S, Melendrez MC, Maljkovic Berry I, Duchêne S, Salje H, Cummings DAT, et al. Understanding dengue virus evolution to support epidemic surveillance and counter-measure development. Infect Genet Evol. 2018;62:279–95. 10.1016/j.meegid.2018.04.032 29704626 PMC6396301

[12] Malisheni M, Khaiboullina SF, Rizvanov AA, Takah N, Murewanhema G, Bates M. Clinical efficacy, safety, and immunogenicity of a live attenuated tetravalent dengue vaccine (CYD-TDV) in children: a systematic review with meta-analysis. Front Immunol. 2017;8:863. 10.3389/fimmu.2017.00863 28824613 PMC5543029

[13] Eltom K, Enan K, El Hussein ARM, Elkhidir IM. Dengue virus infection in sub-Saharan Africa between 2010 and 2020: a systematic review and meta-analysis. Front Cell Infect Microbiol. 2021;11:678945. 10.3389/fcimb.2021.678945 34113579 PMC8186319

[14] Vanneste K, Garlant L, Broeders S, Van Gucht S, Roosens NH. Application of whole genome data for in silico evaluation of primers and probes routinely employed for the detection of viral species by RT-qPCR using dengue virus as a case study. BMC Bioinformatics. 2018;19:312. 10.1186/s12859-018-2313-0 30180800 PMC6123964

[15] Sessions OM, Wilm A, Kamaraj US, Choy MM, Chow A, Chong Y, et al. Analysis of dengue virus genetic diversity during human and mosquito infection reveals genetic constraints. PLoS Negl Trop Dis. 2015;9:e0004044. 10.1371/journal.pntd.0004044 26327586 PMC4556638

[16] Amarasinghe A, Kuritsk JN, Letson GW, Margolis HS. Dengue virus infection in Africa. Emerg Infect Dis. 2011;17:1349–54. 21801609 10.3201/eid1708.101515 PMC3381573

[17] Katzelnick LC, Coloma J, Harris E. Dengue: knowledge gaps, unmet needs, and research priorities. Lancet Infect Dis. 2017;17:e88–100. 10.1016/S1473-3099(16)30473-X 28185868 PMC5967882

[18] Holmes EC, Twiddy SS. The origin, emergence and evolutionary genetics of dengue virus. Infect Genet Evol. 2003;3:19–28. 10.1016/S1567-1348(03)00004-2 12797969

[19] Usme-Ciro JA, Méndez JA, Laiton KD, Páez A. The relevance of dengue virus genotypes surveillance at country level before vaccine approval. Hum Vaccin Immunother. 2014;10:2674–8. 10.4161/hv.29563 25483495 PMC4975057

[20] Gonzalez JP, Du Saussay C, Gautun JC, McCormick JB, Mouchet J. [Dengue in Burkina Faso (ex-Upper Volta): seasonal epidemics in the urban area of Ouagadougou] [in French]. Bull Soc Pathol Exot Filiales. 1985;78:7–14. 3886182

[21] Tarnagda Z, Cissé A, Bicaba BW, Diagbouga S, Sagna T, Ilboudo AK, et al. Dengue Fever in Burkina Faso, 2016. Emerg Infect Dis. 2018;24:170–2. 10.3201/eid2401.170973 29260685 PMC5749475

[22] World Health Organization. Dengue fever—Burkina Faso. 2016 [cited 2021 Aug 25]. https://www.who.int/emergencies/disease-outbreak-news/item/18-november-2016-dengue-burkina-faso-en

[23] World Health Organization. Dengue fever—Burkina Faso. 2017 [cited 2021 Aug 25]. https://www.who.int/emergencies/disease-outbreak-news/item/6-november-2017-dengue-burkina-faso-en

[24] Hashimoto T, Kutsuna S, Maeki T, Tajima S, Takaya S, Katanami Y, et al. A case of dengue fever imported from Burkina Faso to Japan in October 2016. Jpn J Infect Dis. 2017;70:675–7. 10.7883/yoken.JJID.2017.181 28890518

[25] Baronti C, Piorkowski G, Touret F, Charrel R, de Lamballerie X, Nougairede A. Complete coding sequences of two dengue virus type 2 strains isolated from an outbreak in Burkina Faso in 2016. Genome Announc. 2017;5:e00209–17. 10.1128/genomeA.00209-17 28450505 PMC5408103

[26] Santiago GA, Vergne E, Quiles Y, Cosme J, Vazquez J, Medina JF, et al. Analytical and clinical performance of the CDC real time RT-PCR assay for detection and typing of dengue virus. PLoS Negl Trop Dis. 2013;7:e2311. 10.1371/journal.pntd.0002311 23875046 PMC3708876

[27] Johnson BW, Russell BJ, Lanciotti RS. Serotype-specific detection of dengue viruses in a fourplex real-time reverse transcriptase PCR assay. J Clin Microbiol. 2005;43:4977–83. 10.1128/JCM.43.10.4977-4983.2005 16207951 PMC1248506

[28] Rabaa MA, Girerd-Chambaz Y, Duong Thi Hue K, Vu Tuan T, Wills B, Bonaparte M, et al. Genetic epidemiology of dengue viruses in phase III trials of the CYD tetravalent dengue vaccine and implications for efficacy. eLife. 2017;6:e24196. 10.7554/eLife.24196 28871961 PMC5584992

[29] Fourié T, Luciani L, Amrane S, Zandotti C, Leparc-Goffart I, Ninove L, et al. Dengue virus type 1 infection in traveler returning from Benin to France, 2019. Emerg Infect Dis. 2020;26:1946–9. 10.3201/eid2608.200055 32687042 PMC7392436

[30] Santiago GA, Vázquez J, Courtney S, Matías KY, Andersen LE, Colón C, et al. Performance of the Trioplex real-time RT-PCR assay for detection of Zika, dengue, and chikungunya viruses. Nat Commun. 2018;9:1391. 10.1038/s41467-018-03772-1 29643334 PMC5895813

[31] Hadinegoro SR, Arredondo-García JL, Capeding MR, Deseda C, Chotpitayasunondh T, Dietze R, et al.; CYD-TDV Dengue Vaccine Working Group. Efficacy and long-term safety of a dengue vaccine in regions of endemic disease. N Engl J Med. 2015;373:1195–206. 10.1056/NEJMoa1506223 26214039

