# Supplementary material for: Retrospective Genomic Characterization of a 2017 Dengue Virus Outbreak, Burkina Faso
Source: Emerg Infect Dis. 2022 Jun;28(6):1198–210. doi: 10.3201/eid2806.212491 (PMC9155902; doi:10.3201/eid2806.212491)
Supplement: Appendix — Additional information about retrospective genomic characterization of a 2017 dengue virus outbreak, Burkina Faso. [file 21-2491-Techapp-s1.pdf]

Article DOI: https://doi.org/10.3201/eid2806.212491

Retrospective Genomic Characterization of a 2017 Dengue Virus Outbreak, Burkina Faso

Appendix

Supplemental Methods

Next-generation sequencing (NGS) used the KAPA RNA HyperPrep library preparation kit (KAPA Biosystems, MA), followed by the RNA Enrichment component of the TruSeq RNA Exome kit (Illumina, CA) with 76,380 dengue probes (https://github.com/cathrnbp/paper-dengue-2021). Sixty-five samples were barcoded, pooled and sequenced using the 600-cycle Miseq Reagent kits v3 (Illumina, CA) on an Illumina Miseq with a minimum of 2 × 151-bp reads. For data analysis, the sequence of random hexamer associated with read one and the Illumina adaptors were removed from the sequencing reads using Cutadapt v1.9.dev1, and low-quality reads or bases were filtered using Prinseq-lite v0.20.3. Reads were de novo assembled into contigs using Ray2 and aligned to the NCBI database using BLAST. When viral contigs were detected, reads were aligned to a reference genome using Bowtie2 v2.0.6, duplicates were removed with Picard (http://broadinstitute.github.io/picard), and a new consensus was generated using a combination of Samtools v0.1.18 and custom scripts (https://github.com/jtladner/Scripts/blob/master/reference-based_assembly/consensus_fasta.py). Only bases with Phred quality score ≥20 were used in consensus calling, and a minimum of 3× read-depth coverage, in support of the consensus, were required to make a call; positions lacking this depth of coverage were treated as missing.

Detailed Phylogenetics and Molecular Clock Analysis

Dengue virus (DENV) genomes longer than 10,000 bp were obtained from the NIAID Virus Pathogen Database and Analysis Resource at http://www.viprbrc.org (DENV1 n=2671; DENV2 n=2091; DENV3 n=1102) (*1*). To determine specific DENV genotypes, genomes were first aligned using MAFFT v7.427 (*2*). Maximum-likelihood phylogenetic trees were

exhaustively (-slow) estimated using FastTree v2.1 with 5000 Shimodaira-Hasegawa tests to compare alternate topologies (*3*). Subtree's were selected and pruned using Figtree and Inkscape.

For our large scale phylodynamics analysis, all genomes from Africa were retained and approximately 10% of the remaining genomes were randomly subsampled. These subsets and our novel genomes from Burkina Faso were aligned using MAFFT v7.427 (DENV1 n=292; DENV2 n=281; DENV3 n=134). The alignment was manually verified in Geneious Prime 2021.0.3 (https://www.geneious.com) and trimmed to the coding sequence. We inferred the maximum-likelihood phylogeny with RAxML v8.2.12 and visualized the tree with TempEst v1.5.3 (*4*). Clock-likeness was assessed with the root to tip panel and outliers were removed from the alignment and in the case of DENV2, genomes from the same country and year were further randomly down-sampled. Final subset alignments consisted of 202, 265, and 133 genomes for DENV1, 2, and 3, respectively.

Time-calibrated phylogenies were estimated with the MCMC method implemented in BEAST v1.10.4 (*5*). The GTR+Γ nucleotide substitution model and Bayesian Skyride time-aware model were used in six independent chains for 50 million steps. Tracer v1.7.1 was used to check convergence of individual and combined chains after removing 10% burn-in (*6*). Tree files were combined with Logcombiner v1.10.4 and a maximum clade credibility tree was generated using TreeAnnotator v1.10.4.

As much of the historic genomic data for DENV consists of partial genomes, and to ensure our phylogenetic trees were not missing crucial data, we also downloaded all DENV genomes from ViPR (DENV1=10,896; DENV2=8,800; DENV3=5,929). Genomes were split by serotype and aligned with MAFFT. Alignments were manually verified in Geneious Prime 2021.0.3, trimmed to the E-gene sequence, and phylogenetic tree inferred using FastTree v2.1.12.

References

1. Pickett BE, Sadat EL, Zhang Y, Noronha JM, Squires RB, Hunt V, et al. ViPR: an open bioinformatics database and analysis resource for virology research. Nucleic Acids Res. 2012;40:D593–8. PubMed https://doi.org/10.1093/nar/gkr859

2. Katoh K, Standley DM. MAFFT multiple sequence alignment software version 7: improvements in performance and usability. Mol Biol Evol. 2013;30:772–80. PubMed https://doi.org/10.1093/molbev/mst010

3. Price MN, Dehal PS, Arkin AP. FastTree 2—approximately maximum-likelihood trees for large alignments. PLoS One. 2010;5:e9490. PubMed https://doi.org/10.1371/journal.pone.0009490

4. Stamatakis A. RAxML version 8: a tool for phylogenetic analysis and post-analysis of large phylogenies. Bioinformatics. 2014;30:1312–3. PubMed https://doi.org/10.1093/bioinformatics/btu033

5. Suchard MA, Lemey P, Baele G, Ayres DL, Drummond AJ, Rambaut A. Bayesian phylogenetic and phylodynamic data integration using BEAST 1.10. Virus Evol. 2018;4:vey016. PubMed https://doi.org/10.1093/ve/vey016

6. Rambaut A, Drummond AJ, Xie D, Baele G, Suchard MA. Posterior summarization in Bayesian phylogenetics using Tracer 1.7. Syst Biol. 2018;67:901–4. PubMed https://doi.org/10.1093/sysbio/syy032

Appendix Table. Samples subjected to testing and the PCR and serology results

NMIMR Lab ID	Name of site	Triolex rRT-PCR ct	Serology		
			Ag NS1	IgM	IgG
BD-01	Bobo Dioulasso (BD)	Undetected			
BD-02	Bobo Dioulasso (BD)	Undetected			
BD-03	Bobo Dioulasso (BD)	Undetected			
BD-04	Bobo Dioulasso (BD)	Undetected			
BD-05	Bobo Dioulasso (BD)	Undetected			
BD-06	Bobo Dioulasso (BD)	Undetected			
BD-07	Bobo Dioulasso (BD)	Undetected			
BD-08	Bobo Dioulasso (BD)	Undetected			
BD-09	Bobo Dioulasso (BD)	Undetected			
BD-10	Bobo Dioulasso (BD)	Undetected			
BD-11	Bobo Dioulasso (BD)	Undetected			
BD-12	Bobo Dioulasso (BD)	Undetected			
BD-13	Bobo Dioulasso (BD)	Undetected			
BD-14	Bobo Dioulasso (BD)	Undetected			
BD-15	Bobo Dioulasso (BD)	Undetected			
BD-16	Bobo Dioulasso (BD)	Undetected			
BD-17	Bobo Dioulasso (BD)	Undetected			
BD-18	Bobo Dioulasso (BD)	Undetected			
BD-19	Bobo Dioulasso (BD)	Undetected			
BD-20	Bobo Dioulasso (BD)	Undetected			
BD-21	Bobo Dioulasso (BD)	Undetected			
BD-22	Bobo Dioulasso (BD)	Undetected			
BD-23	Bobo Dioulasso (BD)	Undetected			
BD-24	Bobo Dioulasso (BD)	Undetected			
BD-25	Bobo Dioulasso (BD)	Undetected			
BD-26	Bobo Dioulasso (BD)	Undetected			
BD-27	Bobo Dioulasso (BD)	Undetected			
BD-28	Bobo Dioulasso (BD)	Undetected			
BD-29	Bobo Dioulasso (BD)	Undetected			
BD-30	Bobo Dioulasso (BD)	Undetected			
BD-31	Bobo Dioulasso (BD)	Undetected			
BD-32	Bobo Dioulasso (BD)	Undetected			
BD-33	Bobo Dioulasso (BD)	Undetected			
BD-34	Bobo Dioulasso (BD)	Undetected			
BD-35	Bobo Dioulasso (BD)	Undetected			

NMIMR Lab ID	Name of site	Trioplex rRT-PCR ct	Serology		
			Ag NS1	IgM	IgG
BD-36	Bobo Dioulasso (BD)	Undetected			
BD-37	Bobo Dioulasso (BD)	Undetected			
BD-38	Bobo Dioulasso (BD)	Undetected			
BD-39	Bobo Dioulasso (BD)	Undetected			
BD-40	Bobo Dioulasso (BD)	Undetected			
BD-41	Bobo Dioulasso (BD)	Undetected			
BD-42	Bobo Dioulasso (BD)	Undetected			
BD-43	Bobo Dioulasso (BD)	Undetected			
BD-44	Bobo Dioulasso (BD)	Undetected			
BD-45	Bobo Dioulasso (BD)	Undetected			
BD-46	Bobo Dioulasso (BD)	Undetected			
BD-47	Bobo Dioulasso (BD)	Undetected			
BD-48	Bobo Dioulasso (BD)	Undetected			
BD-49	Bobo Dioulasso (BD)	Undetected			
BD-50	Bobo Dioulasso (BD)	Undetected			
BD-51	Bobo Dioulasso (BD)	Undetected			
BD-52	Bobo Dioulasso (BD)	Undetected			
BD-53	Bobo Dioulasso (BD)	Undetected			
BD-54	Bobo Dioulasso (BD)	Undetected			
BD-55	Bobo Dioulasso (BD)	Undetected			
BD-56	Bobo Dioulasso (BD)	Undetected			
BD-57	Bobo Dioulasso (BD)	Undetected			
BD-58	Bobo Dioulasso (BD)	Undetected			
BD-59	Bobo Dioulasso (BD)	Undetected			
BD-60	Bobo Dioulasso (BD)	Undetected			
BD-61	Bobo Dioulasso (BD)	Undetected			
BD-62	Bobo Dioulasso (BD)	Undetected			
BD-63	Bobo Dioulasso (BD)	Undetected			
BD-64	Bobo Dioulasso (BD)	Undetected			
BD-65	Bobo Dioulasso (BD)	Undetected			
BD-66	Bobo Dioulasso (BD)	Undetected			
BD-67	Bobo Dioulasso (BD)	Undetected			
BD-68	Bobo Dioulasso (BD)	Undetected			
BD-69	Bobo Dioulasso (BD)	Undetected			
BD-70	Bobo Dioulasso (BD)	Undetected			
OS-01	Ouoga Secteur (OS)	Undetected			
OS-02	Ouoga Secteur (OS)	Undetected			
OS-03	Ouoga Secteur (OS)	Undetected			
OS-04	Ouoga Secteur (OS)	Undetected			
OS-05	Ouoga Secteur (OS)	Undetected			
OS-06	Ouoga Secteur (OS)	Undetected			
OS-07	Ouoga Secteur (OS)	Undetected			
OS-08	Ouoga Secteur (OS)	Undetected			
OS-09	Ouoga Secteur (OS)	Undetected			
OS-10	Ouoga Secteur (OS)	Undetected			
OS-11	Ouoga Secteur (OS)	Undetected			
OS-12	Ouoga Secteur (OS)	Undetected			
OS-13	Ouoga Secteur (OS)	Undetected			
OS-14	Ouoga Secteur (OS)	Undetected			
OS-15	Ouoga Secteur (OS)	Undetected			
OS-16	Ouoga Secteur (OS)	Undetected			
OS-17	Ouoga Secteur (OS)	Undetected			
OS-18	Ouoga Secteur (OS)	Undetected			
IP-001	Institute Pasteur (IP)	Undetected			
IP-002	Institute Pasteur (IP)	Undetected	Positive		
IP-003	Institute Pasteur (IP)	Undetected			
IP-004	Institute Pasteur (IP)	Undetected			
IP-005	Institute Pasteur (IP)	Undetected			
IP-006	Institute Pasteur (IP)	Undetected			
IP-007	Institute Pasteur (IP)	Undetected			
IP-008	Institute Pasteur (IP)	35.5	Positive		
IP-009	Institute Pasteur (IP)	37.1	Positive		
IP-010	Institute Pasteur (IP)	Undetected	Positive		
IP-011	Institute Pasteur (IP)	Undetected	Positive		
IP-012	Institute Pasteur (IP)	40.6	Positive		
IP-013	Institute Pasteur (IP)	Undetected			
IP-014	Institute Pasteur (IP)	Undetected			
IP-015	Institute Pasteur (IP)	Undetected			

NMIMR Lab ID	Name of site	Triplex rRT-PCR ct	Serology		
			Ag NS1	IgM	IgG
IP-016	Institute Pasteur (IP)	Undetected			
IP-017	Institute Pasteur (IP)	Undetected			
IP-018	Institute Pasteur (IP)	Undetected	Positive		
IP-019	Institute Pasteur (IP)	Undetected	Positive	Positive	Positive
IP-020	Institute Pasteur (IP)	Undetected	Positive		
IP-021	Institute Pasteur (IP)	Undetected			
IP-022	Institute Pasteur (IP)	Undetected			
IP-023	Institute Pasteur (IP)	Undetected			
IP-024	Institute Pasteur (IP)	Undetected			
IP-025	Institute Pasteur (IP)	Undetected			
IP-026	Institute Pasteur (IP)	Undetected			
IP-027	Institute Pasteur (IP)	Undetected			
IP-028	Institute Pasteur (IP)	Undetected		Positive	Positive
IP-029	Institute Pasteur (IP)	37.5	Positive		
IP-030	Institute Pasteur (IP)	Undetected			
IP-031	Institute Pasteur (IP)	Undetected	Positive	Positive	
IP-032	Institute Pasteur (IP)	Undetected		Positive	Positive
IP-033	Institute Pasteur (IP)	Undetected			
IP-034	Institute Pasteur (IP)	Undetected	Positive		Positive
IP-035	Institute Pasteur (IP)	Undetected			
IP-036	Institute Pasteur (IP)	40.6	Positive		
IP-037	Institute Pasteur (IP)	Undetected			
IP-038	Institute Pasteur (IP)	Undetected			
IP-039	Institute Pasteur (IP)	Undetected	Positive		
IP-040	Institute Pasteur (IP)	Undetected			
IP-041	Institute Pasteur (IP)	Undetected			
IP-042	Institute Pasteur (IP)	Undetected			
IP-043	Institute Pasteur (IP)	Undetected			
IP-044	Institute Pasteur (IP)	Undetected			
IP-045	Institute Pasteur (IP)	Undetected			
IP-046	Institute Pasteur (IP)	Undetected	Positive		
IP-047	Institute Pasteur (IP)	Undetected			
IP-048	Institute Pasteur (IP)	Undetected			
IP-049	Institute Pasteur (IP)	Undetected			
IP-050	Institute Pasteur (IP)	Undetected			
IP-051	Institute Pasteur (IP)	Undetected		Positive	Positive
IP-052	Institute Pasteur (IP)	Undetected			
IP-053	Institute Pasteur (IP)	Undetected			
IP-054	Institute Pasteur (IP)	Undetected			
IP-055	Institute Pasteur (IP)	Undetected			
IP-056	Institute Pasteur (IP)	Undetected			
IP-057	Institute Pasteur (IP)	Undetected			
IP-058	Institute Pasteur (IP)	Undetected			
IP-059	Institute Pasteur (IP)	Undetected			
IP-060	Institute Pasteur (IP)	38.7			
IP-061	Institute Pasteur (IP)	Undetected			
IP-062	Institute Pasteur (IP)	Undetected			
IP-063	Institute Pasteur (IP)	Undetected			
IP-064	Institute Pasteur (IP)	Undetected			
IP-065	Institute Pasteur (IP)	Undetected			
IP-066	Institute Pasteur (IP)	Undetected			
IP-067	Institute Pasteur (IP)	Undetected			
IP-068	Institute Pasteur (IP)	Undetected			
IP-069	Institute Pasteur (IP)	Undetected			
IP-070	Institute Pasteur (IP)	Undetected			
IP-071	Institute Pasteur (IP)	Undetected			
IP-072	Institute Pasteur (IP)	Undetected			
IP-073	Institute Pasteur (IP)	Undetected			
IP-074	Institute Pasteur (IP)	Undetected			
IP-075	Institute Pasteur (IP)	Undetected			
IP-076	Institute Pasteur (IP)	Undetected			
IP-077	Institute Pasteur (IP)	Undetected			
IP-078	Institute Pasteur (IP)	Undetected			
IP-079	Institute Pasteur (IP)	Undetected			
IP-080	Institute Pasteur (IP)	Undetected			
IP-081	Institute Pasteur (IP)	Undetected			
IP-082	Institute Pasteur (IP)	Undetected			
IP-083	Institute Pasteur (IP)	Undetected			

NMIMR Lab ID	Name of site	Triodplex rRT-PCR ct	Serology		
			Ag NS1	IgM	IgG
IP-084	Institute Pasteur (IP)	Undetected			
IP-085	Institute Pasteur (IP)	Undetected			
IP-086	Institute Pasteur (IP)	Undetected			
IP-087	Institute Pasteur (IP)	Undetected			
IP-088	Institute Pasteur (IP)	Undetected			
IP-089	Institute Pasteur (IP)	Undetected			
IP-090	Institute Pasteur (IP)	Undetected			
IP-091	Institute Pasteur (IP)	36.0	Positive		
IP-092	Institute Pasteur (IP)	Undetected	Positive	Positive	Positive
IP-093	Institute Pasteur (IP)	Undetected			
IP-094	Institute Pasteur (IP)	Undetected			
IP-095	Institute Pasteur (IP)	Undetected			
IP-096	Institute Pasteur (IP)	Undetected		Positive	Positive
IP-097	Institute Pasteur (IP)	Undetected			
IP-098	Institute Pasteur (IP)	Undetected			
IP-099	Institute Pasteur (IP)	40.3	Positive		
IP-100	Institute Pasteur (IP)	Undetected			
IP-101	Institute Pasteur (IP)	Undetected			
IP-102	Institute Pasteur (IP)	Undetected			
IP-103	Institute Pasteur (IP)	34.6	Positive		
IP-104	Institute Pasteur (IP)	Undetected			
IP-105	Institute Pasteur (IP)	42.3	Positive		
IP-106	Institute Pasteur (IP)	Undetected			
IP-107	Institute Pasteur (IP)	Undetected			
IP-108	Institute Pasteur (IP)	Undetected			
IP-109	Institute Pasteur (IP)	Undetected			
IP-110	Institute Pasteur (IP)	Undetected			
IP-111	Institute Pasteur (IP)	Undetected			
IP-112	Institute Pasteur (IP)	39.5	Positive		
IP-113	Institute Pasteur (IP)	Undetected			
IP-114	Institute Pasteur (IP)	Undetected			
IP-115	Institute Pasteur (IP)	Undetected			
IP-116	Institute Pasteur (IP)	Undetected			
IP-117	Institute Pasteur (IP)	Undetected			
IP-118	Institute Pasteur (IP)	Undetected			
IP-119	Institute Pasteur (IP)	Undetected			
IP-120	Institute Pasteur (IP)	42.2	Positive		
IP-121	Institute Pasteur (IP)	29.0	Positive		
IP-122	Institute Pasteur (IP)	Undetected			
IP-123	Institute Pasteur (IP)	Undetected			
IP-124	Institute Pasteur (IP)	Undetected	Positive	Positive	
IP-125	Institute Pasteur (IP)	Undetected			
IP-126	Institute Pasteur (IP)	Undetected			
IP-127	Institute Pasteur (IP)	38.2			
IP-128	Institute Pasteur (IP)	Undetected			
IP-129	Institute Pasteur (IP)	Undetected			
IP-130	Institute Pasteur (IP)	Undetected			
IP-131	Institute Pasteur (IP)	Undetected			
IP-132	Institute Pasteur (IP)	Undetected			
IP-133	Institute Pasteur (IP)	Undetected			
IP-134	Institute Pasteur (IP)	Undetected			
IP-135	Institute Pasteur (IP)	Undetected			
IP-136	Institute Pasteur (IP)	Undetected		Positive	Positive
IP-137	Institute Pasteur (IP)	Undetected			
IP-138	Institute Pasteur (IP)	Undetected		Positive	Positive
IP-139	Institute Pasteur (IP)	Undetected			
IP-140	Institute Pasteur (IP)	Undetected		Positive	Positive
IP-141	Institute Pasteur (IP)	Undetected			
IP-142	Institute Pasteur (IP)	Undetected			
IP-143	Institute Pasteur (IP)	Undetected			
IP-144	Institute Pasteur (IP)	Undetected			
IP-145	Institute Pasteur (IP)	Undetected			
IP-146	Institute Pasteur (IP)	Undetected	Positive		
IP-147	Institute Pasteur (IP)	Undetected			
IP-148	Institute Pasteur (IP)	Undetected			
IP-149	Institute Pasteur (IP)	Undetected			
IP-150	Institute Pasteur (IP)	Undetected			
IP-151	Institute Pasteur (IP)	Undetected			

NMIMR Lab ID	Name of site	Triplex rRT-PCR ct	Serology		
			Ag NS1	IgM	IgG
IP-152	Institute Pasteur (IP)	Undetected			
IP-153	Institute Pasteur (IP)	33.7	Positive		
IP-154	Institute Pasteur (IP)	39.9	Positive		
IP-155	Institute Pasteur (IP)	Undetected			
IP-156	Institute Pasteur (IP)	Undetected			
IP-157	Institute Pasteur (IP)	Undetected			
IP-158	Institute Pasteur (IP)	Undetected			
IP-159	Institute Pasteur (IP)	32.0	Positive		
IP-160	Institute Pasteur (IP)	Undetected			
IP-161	Institute Pasteur (IP)	Undetected			
IP-162	Institute Pasteur (IP)	Undetected			
IP-163	Institute Pasteur (IP)	Undetected			
IP-164	Institute Pasteur (IP)	Undetected			
IP-165	Institute Pasteur (IP)	Undetected		Positive	Positive
IP-166	Institute Pasteur (IP)	Undetected			
IP-167	Institute Pasteur (IP)	Undetected			
IP-168	Institute Pasteur (IP)	Undetected			
IP-169	Institute Pasteur (IP)	Undetected			
IP-170	Institute Pasteur (IP)	Undetected	Positive	Positive	Positive
IP-171	Institute Pasteur (IP)	33.8	Positive	Positive	Positive
IP-172	Institute Pasteur (IP)	Undetected			
IP-173	Institute Pasteur (IP)	Undetected			
IP-174	Institute Pasteur (IP)	Undetected			
IP-175	Institute Pasteur (IP)	Undetected			
IP-176	Institute Pasteur (IP)	Undetected			
IP-177	Institute Pasteur (IP)	Undetected			
IP-178	Institute Pasteur (IP)	Undetected			
IP-179	Institute Pasteur (IP)	22.5	Positive		
IP-180	Institute Pasteur (IP)	Undetected			
IP-181	Institute Pasteur (IP)	Undetected			
IP-182	Institute Pasteur (IP)	Undetected			
IP-183	Institute Pasteur (IP)	Undetected			
IP-184	Institute Pasteur (IP)	Undetected			
IP-185	Institute Pasteur (IP)	Undetected			
IP-186	Institute Pasteur (IP)	Undetected			
IP-187	Institute Pasteur (IP)	Undetected			
IP-188	Institute Pasteur (IP)	Undetected			
IP-189	Institute Pasteur (IP)	Undetected			
IP-190	Institute Pasteur (IP)	Undetected			
IP-191	Institute Pasteur (IP)	Undetected			
IP-192	Institute Pasteur (IP)	Undetected			
IP-193	Institute Pasteur (IP)	Undetected			
IP-194	Institute Pasteur (IP)	25.2			
IP-195	Institute Pasteur (IP)	41.6			
IP-196	Institute Pasteur (IP)	Undetected			
IP-197	Institute Pasteur (IP)	Undetected			
IP-198	Institute Pasteur (IP)	Undetected			
IP-199	Institute Pasteur (IP)	Undetected			
IP-200	Institute Pasteur (IP)	Undetected			
IP-201	Institute Pasteur (IP)	Undetected			
IP-202	Institute Pasteur (IP)	Undetected			
IP-203	Institute Pasteur (IP)	Undetected			
IP-204	Institute Pasteur (IP)	Undetected			
IP-205	Institute Pasteur (IP)	Undetected			
IP-206	Institute Pasteur (IP)	Undetected			
IP-207	Institute Pasteur (IP)	Undetected			
IP-208	Institute Pasteur (IP)	Undetected			
IP-209	Institute Pasteur (IP)	Undetected			
IP-210	Institute Pasteur (IP)	Undetected			
IP-211	Institute Pasteur (IP)	Undetected			
IP-212	Institute Pasteur (IP)	Undetected			
IP-213	Institute Pasteur (IP)	Undetected			
IP-214	Institute Pasteur (IP)	Undetected			
IP-215	Institute Pasteur (IP)	Undetected			
IP-216	Institute Pasteur (IP)	Undetected			
IP-217	Institute Pasteur (IP)	Undetected			
IP-218	Institute Pasteur (IP)	Undetected			
IP-219	Institute Pasteur (IP)	Undetected			

			Serology		
NMIMR Lab ID	Name of site	Trioplex rRT-PCR ct	Ag NS1	IgM	IgG
IP-220	Institute Pasteur (IP)	Undetected			
IP-221	Institute Pasteur (IP)	Undetected			
IP-222	Institute Pasteur (IP)	Undetected			
IP-223	Institute Pasteur (IP)	Undetected			
IP-224	Institute Pasteur (IP)	Undetected			
IP-225	Institute Pasteur (IP)	Undetected			
IP-226	Institute Pasteur (IP)	25.4			
IP-227	Institute Pasteur (IP)	Undetected			
IP-228	Institute Pasteur (IP)	Undetected			
IP-229	Institute Pasteur (IP)	Undetected			
IP-230	Institute Pasteur (IP)	Undetected			
IP-231	Institute Pasteur (IP)	Undetected			
IP-232	Institute Pasteur (IP)	Undetected			
IP-233	Institute Pasteur (IP)	Undetected			
IP-234	Institute Pasteur (IP)	Undetected			
IP-235	Institute Pasteur (IP)	Undetected			
IP-236	Institute Pasteur (IP)	Undetected			
IP-237	Institute Pasteur (IP)	Undetected			
IP-238	Institute Pasteur (IP)	Undetected			
IP-239	Institute Pasteur (IP)	Undetected			
IP-240	Institute Pasteur (IP)	Undetected			
IP-241	Institute Pasteur (IP)	Undetected			
IP-242	Institute Pasteur (IP)	29.5			
IP-243	Institute Pasteur (IP)	Undetected			
IP-244	Institute Pasteur (IP)	Undetected			
IP-245	Institute Pasteur (IP)	Undetected			
IP-246	Institute Pasteur (IP)	31.1			
IP-247	Institute Pasteur (IP)	Undetected			
IP-248	Institute Pasteur (IP)	Undetected			
IP-249	Institute Pasteur (IP)	Undetected			
IP-250	Institute Pasteur (IP)	Undetected			
IP-251	Institute Pasteur (IP)	Undetected			
IP-252	Institute Pasteur (IP)	Undetected			
IP-253	Institute Pasteur (IP)	Undetected			
IP-254	Institute Pasteur (IP)	Undetected			
IP-255	Institute Pasteur (IP)	Undetected			
IP-256	Institute Pasteur (IP)	Undetected			
IP-257	Institute Pasteur (IP)	Undetected			
IP-258	Institute Pasteur (IP)	Undetected			
IP-259	Institute Pasteur (IP)	Undetected			
IP-260	Institute Pasteur (IP)	Undetected			
IP-261	Institute Pasteur (IP)	Undetected			
IP-262	Institute Pasteur (IP)	Undetected			
IP-263	Institute Pasteur (IP)	Undetected			
IP-264	Institute Pasteur (IP)	Undetected			
IP-265	Institute Pasteur (IP)	Undetected			
IP-266	Institute Pasteur (IP)	Undetected			
IP-267	Institute Pasteur (IP)	19.3			
IP-268	Institute Pasteur (IP)	Undetected			
IP-269	Institute Pasteur (IP)	Undetected			
IP-270	Institute Pasteur (IP)	37.1			
IP-271	Institute Pasteur (IP)	Undetected			
IP-272	Institute Pasteur (IP)	Undetected			
IP-273	Institute Pasteur (IP)	Undetected			
IP-274	Institute Pasteur (IP)	Undetected			
IP-275	Institute Pasteur (IP)	Undetected			
IP-276	Institute Pasteur (IP)	Undetected			
IP-277	Institute Pasteur (IP)	Undetected			
IP-278	Institute Pasteur (IP)	Undetected			
IP-279	Institute Pasteur (IP)	Undetected			
IP-280	Institute Pasteur (IP)	Undetected			
IP-281	Institute Pasteur (IP)	Undetected			
IP-282	Institute Pasteur (IP)	Undetected			
IP-283	Institute Pasteur (IP)	Undetected			
IP-284	Institute Pasteur (IP)	Undetected			
IP-285	Institute Pasteur (IP)	Undetected			
IP-286	Institute Pasteur (IP)	Undetected			
IP-287	Institute Pasteur (IP)	Undetected			

NMIMR Lab ID	Name of site	Trioplex rRT-PCR ct	Serology		
			Ag NS1	IgM	IgG
IP-288	Institute Pasteur (IP)	Undetected			
IP-289	Institute Pasteur (IP)	Undetected			
IP-290	Institute Pasteur (IP)	Undetected			
IP-291	Institute Pasteur (IP)	Undetected			
IP-292	Institute Pasteur (IP)	Undetected			
IP-293	Institute Pasteur (IP)	Undetected			
IP-294	Institute Pasteur (IP)	Undetected			
IP-295	Institute Pasteur (IP)	Undetected			
IP-296	Institute Pasteur (IP)	Undetected			
IP-297	Institute Pasteur (IP)	Undetected			
IP-298	Institute Pasteur (IP)	Undetected	Positive		
IP-299	Institute Pasteur (IP)	Undetected			
IP-300	Institute Pasteur (IP)	Undetected			
IP-301	Institute Pasteur (IP)	Undetected			
IP-302	Institute Pasteur (IP)	Undetected	Positive	Positive	Positive
IP-303	Institute Pasteur (IP)	Undetected			
IP-304	Institute Pasteur (IP)	24.1	Positive		
IP-305	Institute Pasteur (IP)	Undetected			
IP-306	Institute Pasteur (IP)	Undetected			
IP-307	Institute Pasteur (IP)	37.3			
IP-308	Institute Pasteur (IP)	Undetected			
IP-309	Institute Pasteur (IP)	Undetected			
IP-310	Institute Pasteur (IP)	30.3	Positive		Positive
IP-311	Institute Pasteur (IP)	Undetected			
IP-312	Institute Pasteur (IP)	Undetected			
IP-313	Institute Pasteur (IP)	Undetected			
IP-314	Institute Pasteur (IP)	23.8	Positive		
IP-315	Institute Pasteur (IP)	Undetected			
IP-316	Institute Pasteur (IP)	Undetected			
IP-317	Institute Pasteur (IP)	Undetected			
IP-318	Institute Pasteur (IP)	Undetected			
IP-319	Institute Pasteur (IP)	Undetected			
IP-320	Institute Pasteur (IP)	Undetected			
IP-321	Institute Pasteur (IP)	Undetected			
IP-322	Institute Pasteur (IP)	Undetected			
IP-323	Institute Pasteur (IP)	Undetected			
IP-324	Institute Pasteur (IP)	Undetected			
IP 325	Institute Pasteur (IP)	Undetected			
IP 326	Institute Pasteur (IP)	Undetected			Positive
IP 327	Institute Pasteur (IP)	Undetected			
IP 328	Institute Pasteur (IP)	Undetected			
IP 329	Institute Pasteur (IP)	Undetected			Positive
IP 330	Institute Pasteur (IP)	Undetected			
IP 331	Institute Pasteur (IP)	Undetected			
IP 332	Institute Pasteur (IP)	Undetected			
IP 333	Institute Pasteur (IP)	Undetected			
IP 334	Institute Pasteur (IP)	Undetected			
IP 335	Institute Pasteur (IP)	Undetected			
IP 336	Institute Pasteur (IP)	Undetected			
IP 337	Institute Pasteur (IP)	Undetected			
IP 338	Institute Pasteur (IP)	Undetected			
IP 339	Institute Pasteur (IP)	Undetected			
IP 340	Institute Pasteur (IP)	Undetected			
IP 341	Institute Pasteur (IP)	Undetected			
IP 342	Institute Pasteur (IP)	Undetected			
IP 343	Institute Pasteur (IP)	Undetected			
IP 344	Institute Pasteur (IP)	Undetected			Positive
IP 345	Institute Pasteur (IP)	Undetected			
IP 346	Institute Pasteur (IP)	Undetected			
IP 347	Institute Pasteur (IP)	Undetected			
IP 348	Institute Pasteur (IP)	Undetected			
IP 349	Institute Pasteur (IP)	Undetected			
IP 350	Institute Pasteur (IP)	Undetected			
IP 351	Institute Pasteur (IP)	Undetected			Positive
IP 352	Institute Pasteur (IP)	Undetected			
IP 353	Institute Pasteur (IP)	Undetected			Positive
IP 354	Institute Pasteur (IP)	Undetected			
IP 355	Institute Pasteur (IP)	Undetected			

			Serology		
NMIMR Lab ID	Name of site	Trioplex rRT-PCR ct	Ag NS1	IgM	IgG
IP 356	Institute Pasteur (IP)	Undetected			Positive
IP 357	Institute Pasteur (IP)	Undetected			
IP 358	Institute Pasteur (IP)	Undetected			Positive
IP 359	Institute Pasteur (IP)	Undetected			
IP 360	Institute Pasteur (IP)	Undetected			
IP 361	Institute Pasteur (IP)	Undetected			
IP 362	Institute Pasteur (IP)	Undetected			Positive
IP 363	Institute Pasteur (IP)	Undetected			
IP 364	Institute Pasteur (IP)	Undetected			
IP 365	Institute Pasteur (IP)	Undetected			
IP 366	Institute Pasteur (IP)	Undetected			
IP 367	Institute Pasteur (IP)	Undetected			
IP 368	Institute Pasteur (IP)	Undetected			Positive
IP 369	Institute Pasteur (IP)	Undetected		Positive	Positive
IP 370	Institute Pasteur (IP)	Undetected			
IP 371	Institute Pasteur (IP)	Undetected			
IP 372	Institute Pasteur (IP)	Undetected			
IP 373	Institute Pasteur (IP)	Undetected			
IP 374	Institute Pasteur (IP)	Undetected			Positive
IP 375	Institute Pasteur (IP)	Undetected			
IP 376	Institute Pasteur (IP)	Undetected			
IP 377	Institute Pasteur (IP)	Undetected			
IP 378	Institute Pasteur (IP)	Undetected			
IP 379	Institute Pasteur (IP)	Undetected			
IP 380	Institute Pasteur (IP)	Undetected			
IP 381	Institute Pasteur (IP)	Undetected			
IP 382	Institute Pasteur (IP)	Undetected			
IP 383	Institute Pasteur (IP)	Undetected			
IP 384	Institute Pasteur (IP)	Undetected			
IP 385	Institute Pasteur (IP)	Undetected			
IP 386	Institute Pasteur (IP)	Undetected			
IP 387	Institute Pasteur (IP)	Undetected	Positive		
IP 388	Institute Pasteur (IP)	Undetected			
IP 389	Institute Pasteur (IP)	Undetected			
IP 390	Institute Pasteur (IP)	Undetected			
IP 391	Institute Pasteur (IP)	Undetected			
IP 392	Institute Pasteur (IP)	Undetected			
IP 393	Institute Pasteur (IP)	Undetected			
IP 394	Institute Pasteur (IP)	Undetected			
IP 395	Institute Pasteur (IP)	Undetected			
IP 396	Institute Pasteur (IP)	Undetected			
IP 397	Institute Pasteur (IP)	Undetected			
IP 398	Institute Pasteur (IP)	Undetected			
IP 399	Institute Pasteur (IP)	Undetected			
IP 400	Institute Pasteur (IP)	Undetected			
IP 401	Institute Pasteur (IP)	Undetected			
IP 402	Institute Pasteur (IP)	Undetected			
IP 403	Institute Pasteur (IP)	Undetected			
IP 404	Institute Pasteur (IP)	Undetected			
IP 405	Institute Pasteur (IP)	Undetected			
IP 406	Institute Pasteur (IP)	Undetected			
IP 407	Institute Pasteur (IP)	Undetected			
IP 408	Institute Pasteur (IP)	Undetected			
IP 409	Institute Pasteur (IP)	Undetected			
IP 410	Institute Pasteur (IP)	Undetected			
IP 411	Institute Pasteur (IP)	Undetected			
IP 412	Institute Pasteur (IP)	Undetected			
IP 413	Institute Pasteur (IP)	Undetected			
IP 414	Institute Pasteur (IP)	Undetected			
IP 415	Institute Pasteur (IP)	Undetected			
IP 416	Institute Pasteur (IP)	Undetected			
IP 417	Institute Pasteur (IP)	Undetected			
IP 418	Institute Pasteur (IP)	Undetected			
IP 419	Institute Pasteur (IP)	Undetected			
IP 420	Institute Pasteur (IP)	Undetected			
IP 421	Institute Pasteur (IP)	Undetected			
IP 422	Institute Pasteur (IP)	Undetected			
IP 423	Institute Pasteur (IP)	Undetected			

			Serology		
NMIMR Lab ID	Name of site	Trioplex rRT-PCR ct	Ag NS1	IgM	IgG
IP 424	Institute Pasteur (IP)	Undetected			
IP 425	Institute Pasteur (IP)	Undetected			
IP 426	Institute Pasteur (IP)	Undetected			
IP 427	Institute Pasteur (IP)	Undetected			
IP 428	Institute Pasteur (IP)	Undetected			
IP 429	Institute Pasteur (IP)	Undetected			
IP 430	Institute Pasteur (IP)	Undetected			
IP 431	Institute Pasteur (IP)	Undetected			
IP 432	Institute Pasteur (IP)	Undetected			
IP 433	Institute Pasteur (IP)	Undetected			
IP 434	Institute Pasteur (IP)	Undetected			
IP 435	Institute Pasteur (IP)	Undetected			
IP 436	Institute Pasteur (IP)	Undetected			
IP 437	Institute Pasteur (IP)	Undetected			
IP 438	Institute Pasteur (IP)	Undetected			
IP 439	Institute Pasteur (IP)	Undetected			
IP 440	Institute Pasteur (IP)	Undetected			
IP 441	Institute Pasteur (IP)	Undetected			
IP 442	Institute Pasteur (IP)	Undetected			
IP 443	Institute Pasteur (IP)	Undetected			
IP 444	Institute Pasteur (IP)	Undetected			
IP 445	Institute Pasteur (IP)	Undetected			
IP 446	Institute Pasteur (IP)	Undetected			
IP 447	Institute Pasteur (IP)	Undetected			
IP 448	Institute Pasteur (IP)	Undetected			
IP 449	Institute Pasteur (IP)	Undetected			
IP 450	Institute Pasteur (IP)	Undetected			Positive
IP 451	Institute Pasteur (IP)	Undetected			
IP 452	Institute Pasteur (IP)	Undetected			
IP 453	Institute Pasteur (IP)	Undetected			Positive
IP 454	Institute Pasteur (IP)	Undetected			
IP 455	Institute Pasteur (IP)	Undetected			
IP 456	Institute Pasteur (IP)	Undetected			
IP 457	Institute Pasteur (IP)	Undetected			
IP 458	Institute Pasteur (IP)	Undetected			
IP 459	Institute Pasteur (IP)	Undetected			
IP 460	Institute Pasteur (IP)	Undetected			
IP 461	Institute Pasteur (IP)	Undetected			
IP 462	Institute Pasteur (IP)	Undetected			
IP 463	Institute Pasteur (IP)	Undetected			
IP 464	Institute Pasteur (IP)	Undetected			Positive
IP 465	Institute Pasteur (IP)	Undetected			
IP 466	Institute Pasteur (IP)	Undetected			
IP 467	Institute Pasteur (IP)	Undetected			
IP 468	Institute Pasteur (IP)	Undetected			
IP 469	Institute Pasteur (IP)	Undetected			
IP 470	Institute Pasteur (IP)	Undetected			
IP 471	Institute Pasteur (IP)	Undetected			
IP 472	Institute Pasteur (IP)	Undetected			
IP 473	Institute Pasteur (IP)	Undetected			
IP 474	Institute Pasteur (IP)	Undetected			
IP 475	Institute Pasteur (IP)	Undetected			
IP 476	Institute Pasteur (IP)	Undetected			Positive
IP 477	Institute Pasteur (IP)	Undetected			
IP 478	Institute Pasteur (IP)	Undetected			
IP 479	Institute Pasteur (IP)	Undetected			
IP 480	Institute Pasteur (IP)	Undetected			
IP 481	Institute Pasteur (IP)	Undetected			
IP 482	Institute Pasteur (IP)	Undetected			
IP 483	Institute Pasteur (IP)	Undetected			
IP 484	Institute Pasteur (IP)	Undetected			
IP 485	Institute Pasteur (IP)	Undetected			
IP 486	Institute Pasteur (IP)	Undetected			
IP 487	Institute Pasteur (IP)	Undetected			
IP 488	Institute Pasteur (IP)	Undetected			
IP 489	Institute Pasteur (IP)	Undetected			
IP 490	Institute Pasteur (IP)	Undetected			
IP 491	Institute Pasteur (IP)	Undetected			

			Serology		
NMIMR Lab ID	Name of site	Trioplex rRT-PCR ct	Ag NS1	IgM	IgG
IP 492	Institute Pasteur (IP)	Undetected			
IP 493	Institute Pasteur (IP)	Undetected			
IP 494	Institute Pasteur (IP)	41.2	Positive		
IP 495	Institute Pasteur (IP)	Undetected			
IP 496	Institute Pasteur (IP)	Undetected			
IP 497	Institute Pasteur (IP)	Undetected			
IP 498	Institute Pasteur (IP)	Undetected			
IP 499	Institute Pasteur (IP)	Undetected			
IP 500	Institute Pasteur (IP)	Undetected			
IP 501	Institute Pasteur (IP)	Undetected			
IP 502	Institute Pasteur (IP)	Undetected			
IP 503	Institute Pasteur (IP)	Undetected			
IP 504	Institute Pasteur (IP)	Undetected			
IP 505	Institute Pasteur (IP)	Undetected			
IP 506	Institute Pasteur (IP)	Undetected			
IP 507	Institute Pasteur (IP)	Undetected	Positive		
IP 508	Institute Pasteur (IP)	Undetected			
IP 509	Institute Pasteur (IP)	Undetected			
IP 510	Institute Pasteur (IP)	Undetected			
IP 511	Institute Pasteur (IP)	Undetected			
IP 512	Institute Pasteur (IP)	Undetected			
IP 513	Institute Pasteur (IP)	Undetected			
IP 514	Institute Pasteur (IP)	Undetected			
IP 515	Institute Pasteur (IP)	Undetected			
IP 516	Institute Pasteur (IP)	Undetected			
IP 517	Institute Pasteur (IP)	Undetected			
IP 518	Institute Pasteur (IP)	Undetected			
IP 519	Institute Pasteur (IP)	Undetected			
IP 520	Institute Pasteur (IP)	Undetected			
IP 521	Institute Pasteur (IP)	Undetected			
IP 522	Institute Pasteur (IP)	Undetected			
IP 523	Institute Pasteur (IP)	Undetected			
IP 524	Institute Pasteur (IP)	Undetected			
IP 525	Institute Pasteur (IP)	Undetected			
IP 526	Institute Pasteur (IP)	Undetected			
IP 527	Institute Pasteur (IP)	Undetected			
IP 528	Institute Pasteur (IP)	Undetected			
IP 529	Institute Pasteur (IP)	Undetected			
IP 530	Institute Pasteur (IP)	Undetected			
IP 531	Institute Pasteur (IP)	Undetected			
IP 532	Institute Pasteur (IP)	Undetected			
IP 533	Institute Pasteur (IP)	Undetected	Positive		
IP 534	Institute Pasteur (IP)	Undetected			
IP 535	Institute Pasteur (IP)	Undetected			
IP 536	Institute Pasteur (IP)	Undetected		Positive	Positive
IP 537	Institute Pasteur (IP)	Undetected			
IP 538	Institute Pasteur (IP)	Undetected	Positive		
IP 539	Institute Pasteur (IP)	Undetected			
IP 540	Institute Pasteur (IP)	Undetected	Positive		
IP 541	Institute Pasteur (IP)	Undetected			
IP 542	Institute Pasteur (IP)	Undetected			
IP 543	Institute Pasteur (IP)	Undetected			
IP 544	Institute Pasteur (IP)	Undetected			Positive
IP 545	Institute Pasteur (IP)	Undetected			
IP 546	Institute Pasteur (IP)	Undetected			
IP 547	Institute Pasteur (IP)	Undetected			
IP 548	Institute Pasteur (IP)	Undetected			
IP 549	Institute Pasteur (IP)	Undetected			
IP 550	Institute Pasteur (IP)	Undetected			
IP 551	Institute Pasteur (IP)	Undetected			
IP 552	Institute Pasteur (IP)	Undetected			
IP 553	Institute Pasteur (IP)	Undetected			
IP 554	Institute Pasteur (IP)	Undetected			
IP 555	Institute Pasteur (IP)	Undetected			
IP 556	Institute Pasteur (IP)	Undetected			
IP 557	Institute Pasteur (IP)	Undetected			
IP 558	Institute Pasteur (IP)	Undetected			
IP 559	Institute Pasteur (IP)	Undetected			

NMIMR Lab ID	Name of site	Triplex rRT-PCR ct	Serology		
			Ag NS1	IgM	IgG
IP 560	Institute Pasteur (IP)	Undetected	Positive		
IP 561	Institute Pasteur (IP)	Undetected			
IP 562	Institute Pasteur (IP)	Undetected			
IP 563	Institute Pasteur (IP)	Undetected			
IP 564	Institute Pasteur (IP)	Undetected			
IP 565	Institute Pasteur (IP)	Undetected			
IP 566	Institute Pasteur (IP)	Undetected			
IP 567	Institute Pasteur (IP)	Undetected			
IP 568	Institute Pasteur (IP)	Undetected			
IP 569	Institute Pasteur (IP)	Undetected			
IP 570	Institute Pasteur (IP)	Undetected			
IP 571	Institute Pasteur (IP)	Undetected			
IP 572	Institute Pasteur (IP)	Undetected			
IP 573	Institute Pasteur (IP)	Undetected			
IP 574	Institute Pasteur (IP)	Undetected			
IP 575	Institute Pasteur (IP)	Undetected			
IP 576	Institute Pasteur (IP)	Undetected			
IP 577	Institute Pasteur (IP)	Undetected			
IP 578	Institute Pasteur (IP)	Undetected			Positive
IP 579	Institute Pasteur (IP)	Undetected			
IP 580	Institute Pasteur (IP)	Undetected			
IP 581	Institute Pasteur (IP)	Undetected			
IP 582	Institute Pasteur (IP)	Undetected			
IP 583	Institute Pasteur (IP)	Undetected			
IP 584	Institute Pasteur (IP)	Undetected			
IP 585	Institute Pasteur (IP)	Undetected			Positive
IP 586	Institute Pasteur (IP)	Undetected			
IP 587	Institute Pasteur (IP)	Undetected			
IP 588	Institute Pasteur (IP)	Undetected			
IP 589	Institute Pasteur (IP)	Undetected			
IP 590	Institute Pasteur (IP)	Undetected			
IP 591	Institute Pasteur (IP)	Undetected			
IP 592	Institute Pasteur (IP)	Undetected			
IP 593	Institute Pasteur (IP)	Undetected			
IP 594	Institute Pasteur (IP)	Undetected			
IP 595	Institute Pasteur (IP)	Undetected			
IP 596	Institute Pasteur (IP)	Undetected			
IP 597	Institute Pasteur (IP)	Undetected			
IP 598	Institute Pasteur (IP)	Undetected			
IP 599	Institute Pasteur (IP)	Undetected			
IP 600	Institute Pasteur (IP)	Undetected			
IP 601	Institute Pasteur (IP)	Undetected			
IP 602	Institute Pasteur (IP)	Undetected			
IP 603	Institute Pasteur (IP)	Undetected			
IP 604	Institute Pasteur (IP)	Undetected			
IP 605	Institute Pasteur (IP)	Undetected			
IP 606	Institute Pasteur (IP)	Undetected			
IP 607	Institute Pasteur (IP)	Undetected			
IP 608	Institute Pasteur (IP)	Undetected			
IP 609	Institute Pasteur (IP)	Undetected			
IP 610	Institute Pasteur (IP)	Undetected			
IP 611	Institute Pasteur (IP)	Undetected			
IP 612	Institute Pasteur (IP)	Undetected			
IP 613	Institute Pasteur (IP)	Undetected			
IP 614	Institute Pasteur (IP)	Undetected			
IP 615	Institute Pasteur (IP)	Undetected			
IP 616	Institute Pasteur (IP)	Undetected			
IP 617	Institute Pasteur (IP)	Undetected			
IP 618	Institute Pasteur (IP)	Undetected			
IP 619	Institute Pasteur (IP)	Undetected			
IP 620	Institute Pasteur (IP)	Undetected			Positive
IP 621	Institute Pasteur (IP)	Undetected			
IP 622	Institute Pasteur (IP)	Undetected			
IP 623	Institute Pasteur (IP)	Undetected			
IP 624	Institute Pasteur (IP)	Undetected			
IP 625	Institute Pasteur (IP)	Undetected			
IP 626	Institute Pasteur (IP)	Undetected			
IP 627	Institute Pasteur (IP)	Undetected			

			Serology		
NMIMR Lab ID	Name of site	Trioplex rRT-PCR ct	Ag NS1	IgM	IgG
IP 628	Institute Pasteur (IP)	Undetected			
IP 629	Institute Pasteur (IP)	Undetected			
IP 630	Institute Pasteur (IP)	Undetected			
IP 631	Institute Pasteur (IP)	Undetected			
IP 632	Institute Pasteur (IP)	Undetected			
IP 633	Institute Pasteur (IP)	Undetected			
IP 634	Institute Pasteur (IP)	Undetected			
IP 635	Institute Pasteur (IP)	Undetected			
IP 636	Institute Pasteur (IP)	Undetected			
IP 637	Institute Pasteur (IP)	Undetected			
IP 638	Institute Pasteur (IP)	Undetected			
IP 639	Institute Pasteur (IP)	Undetected			
IP 640	Institute Pasteur (IP)	Undetected			
IP 641	Institute Pasteur (IP)	Undetected		Positive	Positive
IP 642	Institute Pasteur (IP)	Undetected			
IP 643	Institute Pasteur (IP)	Undetected			
IP 644	Institute Pasteur (IP)	Undetected			
IP 645	Institute Pasteur (IP)	Undetected			
IP 646	Institute Pasteur (IP)	Undetected			
IP 647	Institute Pasteur (IP)	Undetected			
IP 648	Institute Pasteur (IP)	Undetected			
IP 649	Institute Pasteur (IP)	Undetected			
IP 650	Institute Pasteur (IP)	Undetected			
IP 651	Institute Pasteur (IP)	Undetected			
IP 652	Institute Pasteur (IP)	Undetected			
IP 653	Institute Pasteur (IP)	Undetected			
IP 654	Institute Pasteur (IP)	Undetected			
IP 655	Institute Pasteur (IP)	Undetected			
IP 656	Institute Pasteur (IP)	Undetected			
IP 657	Institute Pasteur (IP)	Undetected			
IP 658	Institute Pasteur (IP)	Undetected			
IP 659	Institute Pasteur (IP)	Undetected			
IP 660	Institute Pasteur (IP)	Undetected			
IP 661	Institute Pasteur (IP)	Undetected			
IP 662	Institute Pasteur (IP)	Undetected			
IP 663	Institute Pasteur (IP)	Undetected			
IP 664	Institute Pasteur (IP)	Undetected			
IP 665	Institute Pasteur (IP)	Undetected			
IP 666	Institute Pasteur (IP)	Undetected	Positive		
IP 667	Institute Pasteur (IP)	Undetected			
IP 668	Institute Pasteur (IP)	Undetected			
IP 669	Institute Pasteur (IP)	Undetected			Positive
IP 670	Institute Pasteur (IP)	Undetected			
IP 671	Institute Pasteur (IP)	Undetected			
IP 672	Institute Pasteur (IP)	Undetected			
IP 673	Institute Pasteur (IP)	Undetected			
IP 674	Institute Pasteur (IP)	Undetected			Positive
IP 675	Institute Pasteur (IP)	Undetected			
IP 676	Institute Pasteur (IP)	Undetected			
IP 677	Institute Pasteur (IP)	Undetected			Positive
IP 678	Institute Pasteur (IP)	Undetected			
IP 679	Institute Pasteur (IP)	Undetected			
IP 680	Institute Pasteur (IP)	Undetected			
IP 681	Institute Pasteur (IP)	Undetected			
IP 682	Institute Pasteur (IP)	Undetected			
IP 683	Institute Pasteur (IP)	Undetected			
IP 684	Institute Pasteur (IP)	Undetected			
IP 685	Institute Pasteur (IP)	Undetected			
IP 686	Institute Pasteur (IP)	Undetected			
IP 687	Institute Pasteur (IP)	Undetected			
IP 688	Institute Pasteur (IP)	Undetected			
IP 689	Institute Pasteur (IP)	Undetected			
IP 690	Institute Pasteur (IP)	Undetected			
IP 691	Institute Pasteur (IP)	Undetected			
IP 692	Institute Pasteur (IP)	Undetected			
IP 693	Institute Pasteur (IP)	Undetected			
IP 694	Institute Pasteur (IP)	Undetected			
IP 695	Institute Pasteur (IP)	Undetected			

NMIMR Lab ID	Name of site	Trioplex rRT-PCR ct	Serology		
			Ag NS1	IgM	IgG
IP 696	Institute Pasteur (IP)	Undetected			
IP 697	Institute Pasteur (IP)	Undetected			
IP 698	Institute Pasteur (IP)	Undetected			
IP 699	Institute Pasteur (IP)	Undetected			
IP 700	Institute Pasteur (IP)	Undetected			
IP 701	Institute Pasteur (IP)	Undetected			
IP 702	Institute Pasteur (IP)	Undetected			
IP 703	Institute Pasteur (IP)	Undetected			

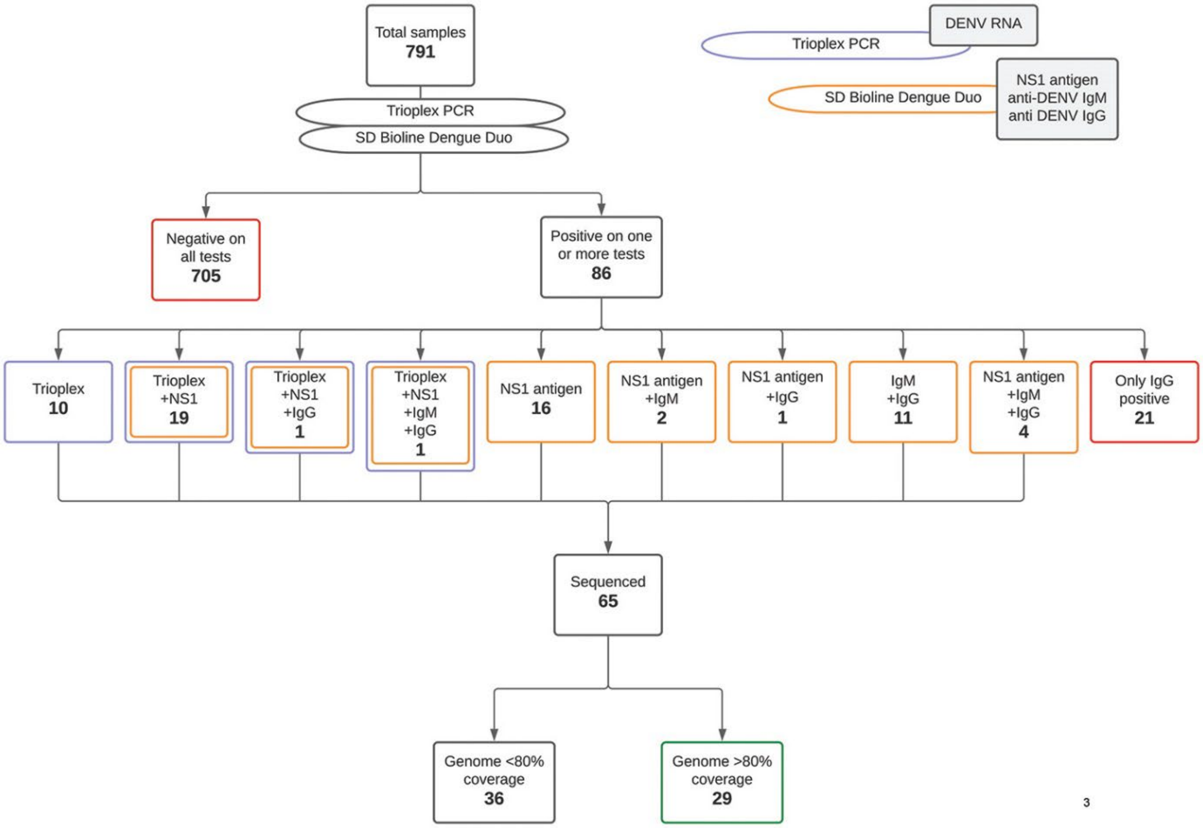

Appendix Figure 1. Sample testing workflow.

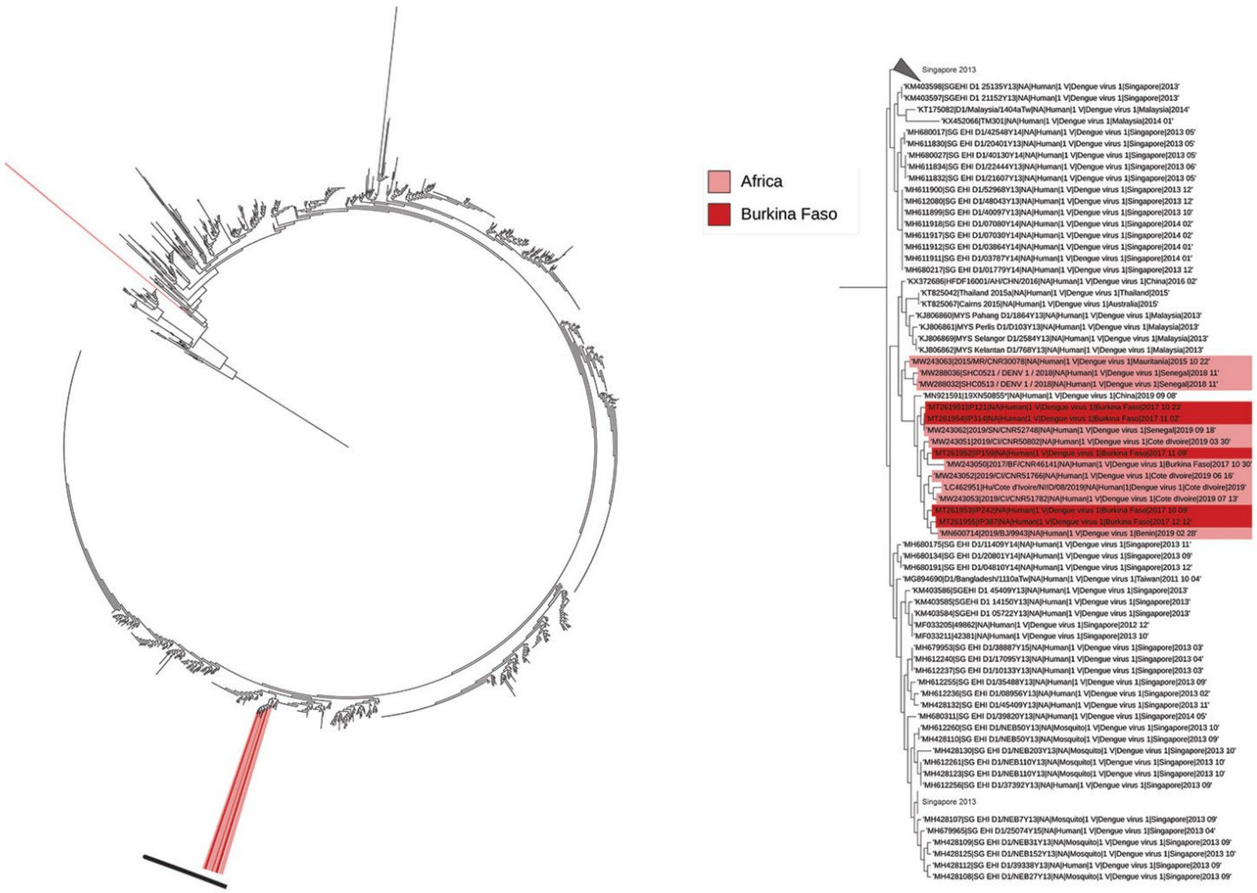

Appendix Figure 2. Dengue virus serotype 1 phylogenetic tree inferred from all available DENV-1 sequences with E-gene coverage. Presented circular tree is a subtree including only genotype V. The tree on the right is a further subtree as indicated by the black bar. Genomes highlighted in pink originated from countries in Africa and those in red from Burkina Faso. When all known sequences are included in the tree, conclusions from the whole genome analysis are still supported.

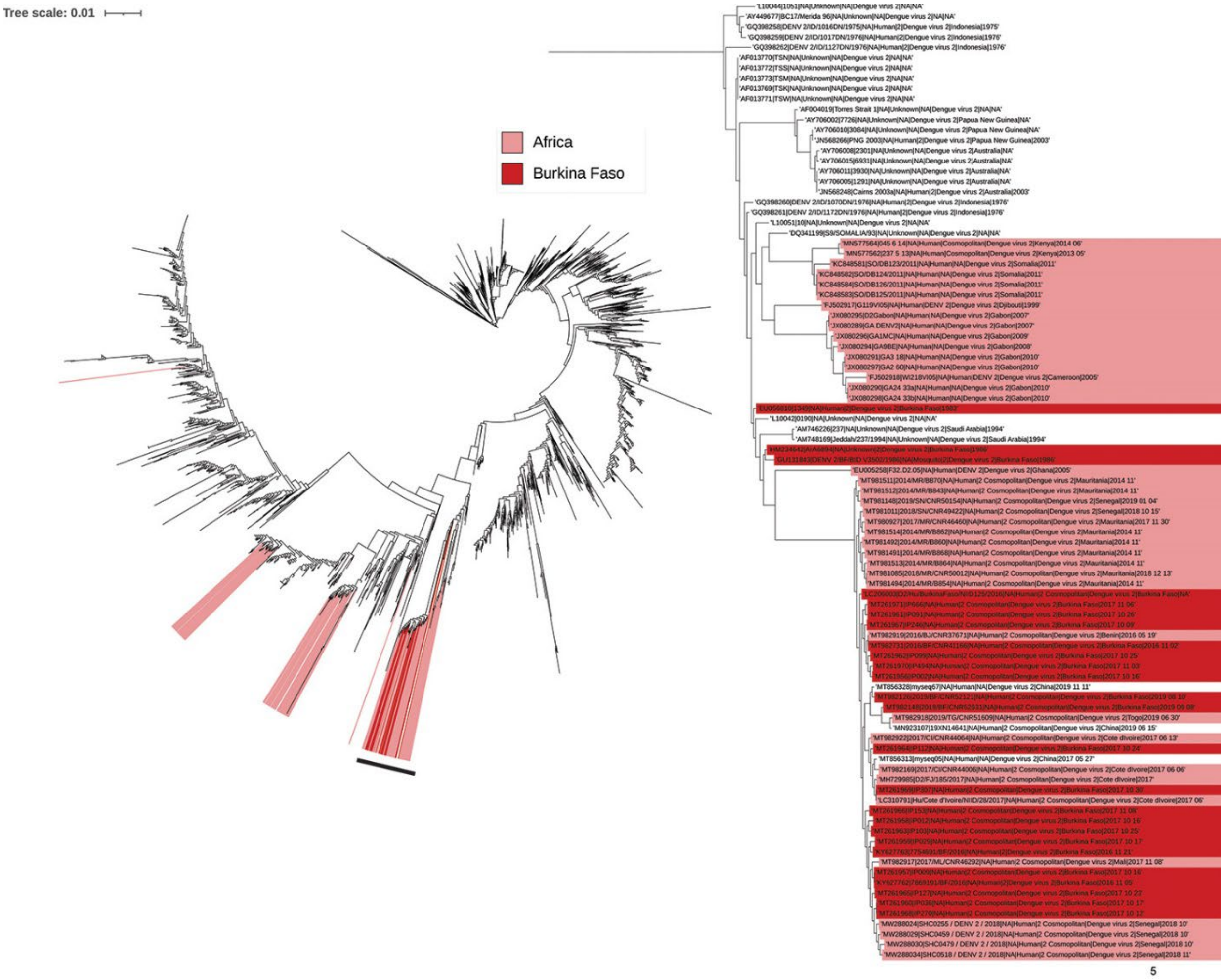

Appendix Figure 3. Dengue virus serotype 2 phylogenetic tree inferred from all available DENV-2 sequences with E-gene coverage. Presented circular tree is a subtree including only genotype Cosmopolitan. The tree on the right is a further subtree as indicated by the black bar. Genomes highlighted in pink originated from countries in Africa and those in red from Burkina Faso. When all know sequences are included in the tree, conclusions from the whole genome analysis are still supported.

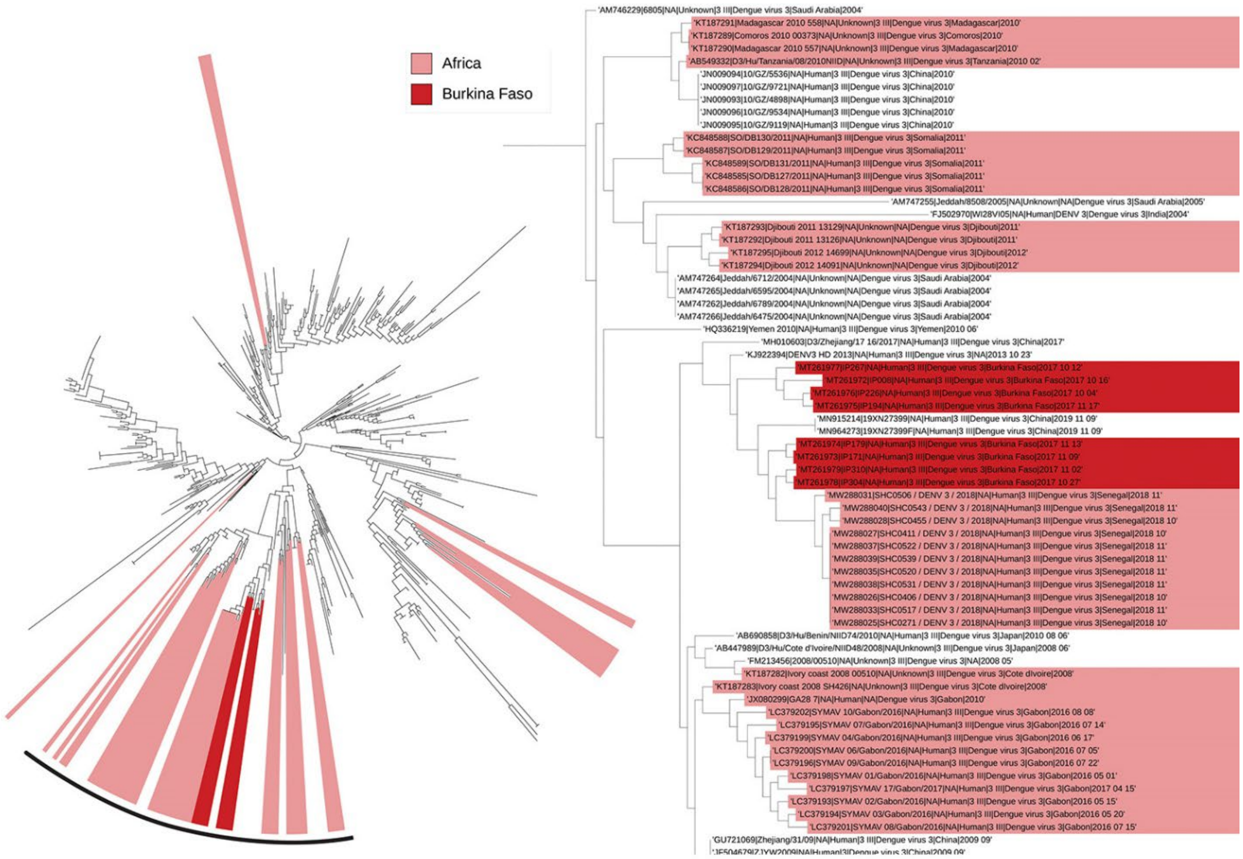

Appendix Figure 4. Dengue virus serotype 3 phylogenetic tree inferred from all available DENV-3 sequences with E-gene coverage. Presented circular tree is a subtree including only genotype III. The tree on the right is a further subtree as indicated by the black bar. Genomes highlighted in pink originated from countries in Africa and those in red from Burkina Faso. When all known sequences are included in the tree, conclusions from the whole genome analysis are still supported.

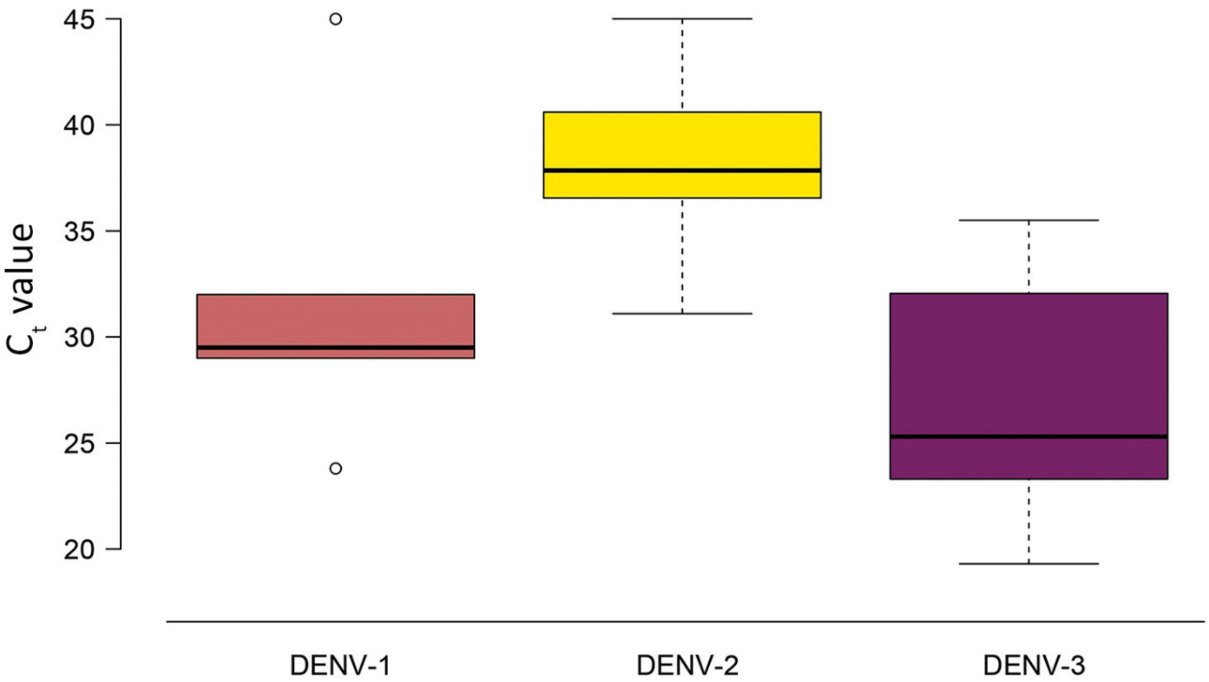

Appendix Figure 5. Box plots of Trioplex rRT-PCR Ct values for the 29 genomes with over 85% coverage from the 2017 Burkina Faso dengue virus outbreak. Undetected samples were designated a Ct of 45. The Trioplex rRT-PCR exhibits a higher median Ct against DENV-2, than DENV-1 or −3, indicating lower sensitivity toward DENV-2 sequences. Of note for DENV1, only 5 values were available therefore the upper and lower values are represented by points.

		DENV1	DENV2	DENV3	Gene target
**Johnson et al.**	*Forward*	0	1	1	**DENV-1: NS5 gene**
	Reverse	1	2	0	**DENV-2: E gene**
	Probe	0	3	0	**DENV-3: prM gene**
**CDC-DENV-1-4**	*Forward*	0	0	0	**DENV-1: NS5 gene**
	Reverse	2	1	0	**DENV-2: E gene**
	Probe	0	2	0	**DENV-3: prM gene**
**Trioplex**	*Forward*	0	1	0	**5' UTR/C gene**
	Reverse1	0	1	0	
	Probe	0	0	0	

Appendix Figure 6. Heatmap indicating the number of mismatches between dengue virus molecular diagnostics and circulating dengue virus genomes. Burkina Faso dengue virus outbreak genomes from 2017 were compared to the Trioplex, CDC-DENV-1–4, and Johnson et al. primer-probe sequences within the primer-probe binding sites. All three diagnostics have mismatches against the 2017 Burkina Faso dengue virus outbreak genomes, especially against DENV-2 genomes.

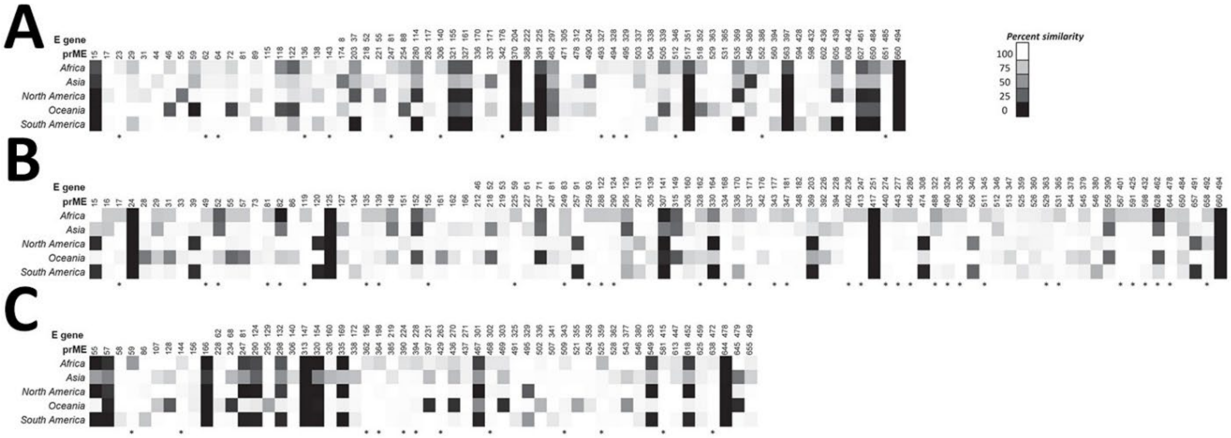

Appendix Figure 7. Conserved site analysis between the Dengvaxia® (CYD-TDV) vaccine and all A) DENV-1, B) DENV-2, and C) DENV-3 genomes trimmed to the prM and E gene region, and split by continent. Only amino acid positions with more than 5% divergence in at least one continent are shown for clarity. The darker the color, the greater the proportion of DENV genomes within that continent that are divergent from the vaccine amino acid at that position. Amino acid positions where African genomes are the most divergent are marked with an asterisk.

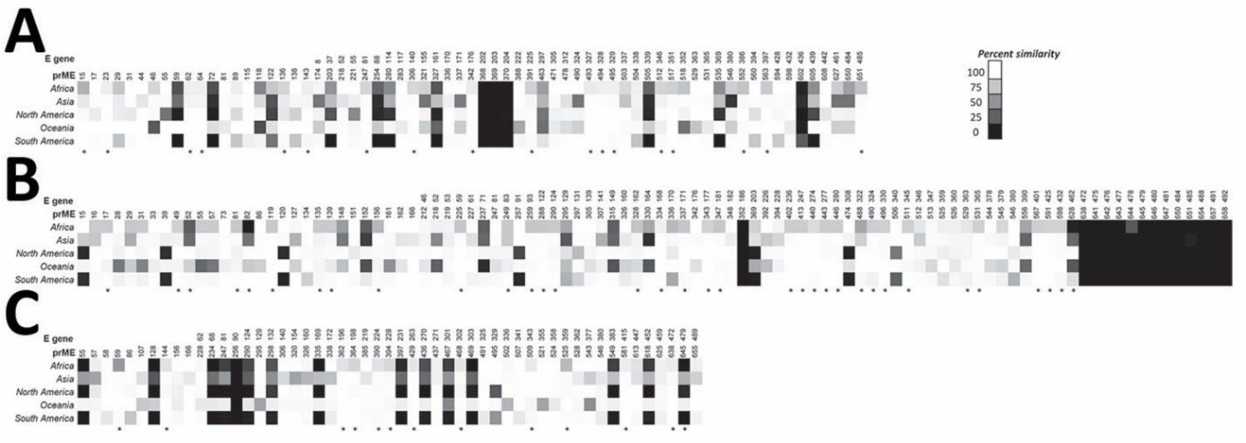

Appendix Figure 8. Conserved site analysis between the TetraVax-DV-TV003 (TV003) vaccine and all A) DENV-1, B) DENV-2, and C) DENV-3 genomes trimmed to the prM and E gene region, and split by continent. Only amino acid positions with more than 5% divergence in at least one continent are shown for clarity. The darker the color, the greater the proportion of DENV genomes within that continent that are divergent from the vaccine amino acid at that position. Amino acid positions where African genomes are the most divergent are marked with an asterisk.
